# TREM-2 is a sensor and activator of T cell response in SARS-CoV-2 infection

**DOI:** 10.1126/sciadv.abi6802

**Published:** 2021-12-08

**Authors:** Yongjian Wu, Manni Wang, Huan Yin, Siqi Ming, Xingyu Li, Guanmin Jiang, Ye Liu, Peihui Wang, Guangde Zhou, Lei Liu, Sitang Gong, Haibo Zhou, Hong Shan, Xi Huang

**Affiliations:** 1 Center for Infection and Immunity, The Fifth Affiliated Hospital, Sun Yat-sen University, Zhuhai, Guangdong Province 519000, China.; 2 Guangdong Provincial Engineering Research Center of Molecular Imaging, Guangdong Provincial Key Laboratory of Biomedical Imaging, and Department of Interventional Medicine, The Fifth Affiliated Hospital, Sun Yat-sen University, Zhuhai, Guangdong Province 519000, China.; 3 Southern Marine Science and Engineering Guangdong Laboratory, Zhuhai, Guangdong Province 519000, China.; 4 Department of Gastroenterology, Guangzhou Women and Children’s Medical Center, Guangzhou Institute of Pediatrics, Guangzhou Medical University, Guangzhou, Guangdong Province 510623, China.; 5 The Sixth Affiliated Hospital of Guangzhou Medical University, Qingyuan People’s Hospital, Qingyuan, Guangdong Province 511518, China.; 6 National Clinical Research Center for Infectious Disease, Shenzhen Third People’s Hospital, The Second Affiliated Hospital of Southern University of Science and Technology, Shenzhen, Guangdong Province 518112, China.; 7 Department of Clinical Laboratory, The Fifth Affiliated Hospital of Sun Yat-sen University, Zhuhai, Guangdong Province 519000, China.; 8 Advanced Medical Research Institute, Cheeloo College of Medicine, Shandong University, Jinan, Shandong Province 250012, China.

## Abstract

Limited understanding of T cell responses against severe acute respiratory syndrome coronavirus 2 (SARS-CoV-2) has impeded vaccine development and drug discovery for coronavirus disease 2019 (COVID-19). We found that triggering receptor expressed on myeloid cells 2 (TREM-2) was induced in T cells in the blood and lungs of patients with COVID-19. After binding to SARS-CoV-2 membrane (M) protein through its immunoglobulin domain, TREM-2 then activated the CD3ζ/ZAP70 complex, leading to STAT1 phosphorylation and T-bet transcription. In vitro stimulation with M protein-reconstituted pseudovirus or recombinant M protein, and TREM-2 promoted the T helper cell 1 (T_H_1) cytokines interferon-γ and tumor necrosis factor. In vivo infection of CD4–TREM-2 conditional knockout mice with murine coronavirus mouse hepatitis virus A-59 showed that intrinsic TREM-2 in T cells enhanced T_H_1 response and viral clearance, thus aggravating lung destruction. These findings demonstrate a previously unidentified role for TREM-2 in SARS-CoV-2 infection, and suggest potential strategies for drug discovery and clinical management of COVID-19.

## Teaser

TREM-2 recognizes SARS-CoV-2 membrane protein and activates CD3ζ/ZAP70/STAT1 signal pathway to induce antiviral T_H_1 response.

## INTRODUCTION

The world changed in December 2019 with the emergence of a new zoonotic pathogen, severe acute respiratory syndrome coronavirus 2 (SARS-CoV-2), which causes a variety of clinical syndromes collectively termed coronavirus disease 2019 (COVID-19). Severe COVID-19 could lead to respiratory failure, septic shock, and, ultimately, death (*1*–*3*). The current severe SARS-CoV-2 pandemic highlights the clinical consequences of immune dysregulation in host antiviral immunity (*4*). T lymphocytes play a vital role in cell-mediated and humoral immunity. During virus infection, CD8^+^ cytotoxic T cells (CTLs) are capable of secreting an array of molecules such as perforin, granzymes, and interferon-γ (IFN-γ) to eradicate viruses from the host (*5*). At the same time, CD4^+^ T helper cells (T_H_) can assist CTLs and cytotoxic B cells and enhance their abilities to clear virus (*6*). A beneficial role of T cells in combating COVID-19 would be in line with observations that CD4^+^ and CD8^+^ T cells are protective against the closely related SARS-CoVs (*7*–*9*). Only a limited number of studies have characterized the SARS-CoV-2–specific T cell responses in patients with COVID-19 (*10*–*12*). SARS-CoV-2 spike (S), membrane (M), and nucleocapsid (N) peptide–treated peripheral blood mononuclear cells (PBMCs) up-regulated IFN-γ but not interleukin-4 (IL-4) or IL-17, suggesting that a T_H_1 response was induced in COVID-19 (*11*, *12*). The study of phenotype based on CD45RA and CCR7 suggested SARS-CoV-2–specific T cells to be more of the effector memory phenotype (*12*). Subsequently, T cell–mediated immune responses and inflammation are proved to be critical for host elimination against SARS-CoV-2 (*13*). These studies indicated that T cells play a key role in antivirus immunity in COVID-19. Thus, it is critical to investigate the regulatory mechanism of T cell functions in patients with COVID-19.

In the initiation of innate immune responses against pathogens, pattern recognition receptors (PRRs) have an essential role in recognizing specific components of microorganisms and triggering responses that eliminate the invading microorganisms (*14*). These PRRs include membrane-bound C-type lectin receptors, cytosolic proteins such as Toll-like receptors (TLRs), nucleotide oligomerization domain (NOD)–like receptors, retinoic acid-inducible gene I (RIG-I)–like receptors, and unidentified proteins that mediate sensing of microbial components (*14*). The pathogen-associated molecular patterns (PAMPs) and damage-associated molecular patterns induce inflammatory T cells either indirectly, through the induction of proinflammatory cytokine production by innate immune cells, or directly, by binding to PRRs on T cells (*15*). T cells also express PRRs such as TLRs, and evidence is emerging that TLR signaling in T cells can promote cytokine secretion or regulate their function (*16*, *17*).

Triggering receptors expressed on myeloid cells (TREMs) belong to a family of innate immune receptors that broadly express in monocytes, macrophages, and dendritic cells (*18*). The most well-characterized members of the TREM family are TREM-1 and TREM-2, which signal through the same adaptor molecule, DNAX activating protein of 12 kDa (DAP12), but execute distinct functions on inflammatory modulation. In myeloid cells, TREM-1 amplifies TLR signaling and host inflammation (*19*), whereas TREM-2 inhibits the secretion of proinflammatory cytokines induced by the TLR and Fc receptor response (*20*). Although substantive evidence has demonstrated the anti-inflammatory properties of TREM-2 in innate immune cells, in vivo studies have shown contradictory effects of TREM-2 in modulating infectious and inflammatory diseases (*21*–*23*). On one hand, TREM-2 fine-tunes inflammatory responses in a murine model of sepsis induced by Gram-negative bacteria. Mice lacking TREM-2 exhibited heightened liver damage and inflammation in a chemical reagent–induced liver injury model (*21*). On the other hand, TREM-2 deficiency has been shown to attentuate the inflammatory response and restrict organ damage and mortality induced by *Burkholderia pseudomallei* infection (*22*). Knockout of TREM-2 also inhibits neuroinflammation and protects against neurodegeneration in a mouse model of tauopathy (*23*). These contradictory reports indicate that TREM-2 may play a distinct role in other immune cells such as lymphocytes that do not have myeloid lineage. Our previous study demonstrated that TREM-1 is highly expressed in Vδ2 T cells from patients with active pulmonary tuberculosis (TB) and that TREM-1 promotes the antigen-presenting capability of Vδ2 T cells (*24*). It is reported that TREM-like transcript 2 (TLT2) is constitutively expressed on CD8^+^ T cells and enhances IL-2 and IFN-γ production (*25*). Moreover, TREM-2 expression was induced in peripheral blood CD4^+^ and CD8^+^ T cells of patients with TB (*26*). Several studies have reported that TREM-2 exhibits distinct roles in viral infection (*27*–*29*). However, the role of TREM-2 in SARS-CoV-2 infection remains uninvestigated.

In this study, we identified TREM-2 as a modulator expressed in T cells during SARS-CoV-2 infection. TREM-2 was up-regulated in periphery and lung-infiltrating T cells from patients with COVID-19. TREM-2^+^ T cells of patients with COVID-19 was positively correlated with clinical indicators of severe COVID-19 and displayed activation and effector memory phenotype. TREM-2 bound to SARS-CoV-2 M protein TREM-2 and interacted with T cell receptor (TCR) subunit CD3ζ and kinase ζ-chain associated protein of 70 kDa (ZAP70). SARS-CoV-2 M protein–reconstituted pseudovirus induced the phosphorylation of CD3ζ, ZAP70, and STAT1 (signal transducers and activators of transcription 1), as well as T-bet expression in TREM-2^+^ T cells. TREM-2 enhanced proinflammatory T_H_1 cytokines IFN-γ and tumor necrosis factor (TNF) produced by T cells upon SARS-CoV-2 M protein stimulation. Furthermore, CD4-specific conditional TREM-2 knockout (CD4–TREM-2 cKO) mice were used to generate the mouse hepatitis virus A-59 (MHV-A59) intranasally inoculated model. The in vitro coronavirus infection model exhibited lower expression of IFN-γ and TNF, together with higher viral load and lung destruction in TREM-2 KO mice compared with wild-type (WT) control, suggesting that TREM-2 is required in T cell–mediated immune defense and inflammation. Our results thus provide a preliminary demonstration of a TREM-2–mediated T cell response, suggesting potential therapeutic targets for infectious and inflammatory diseases.

## RESULTS

### TREM-2 expression was increased on T cells in patients with COVID-19

**Fig. 1.**
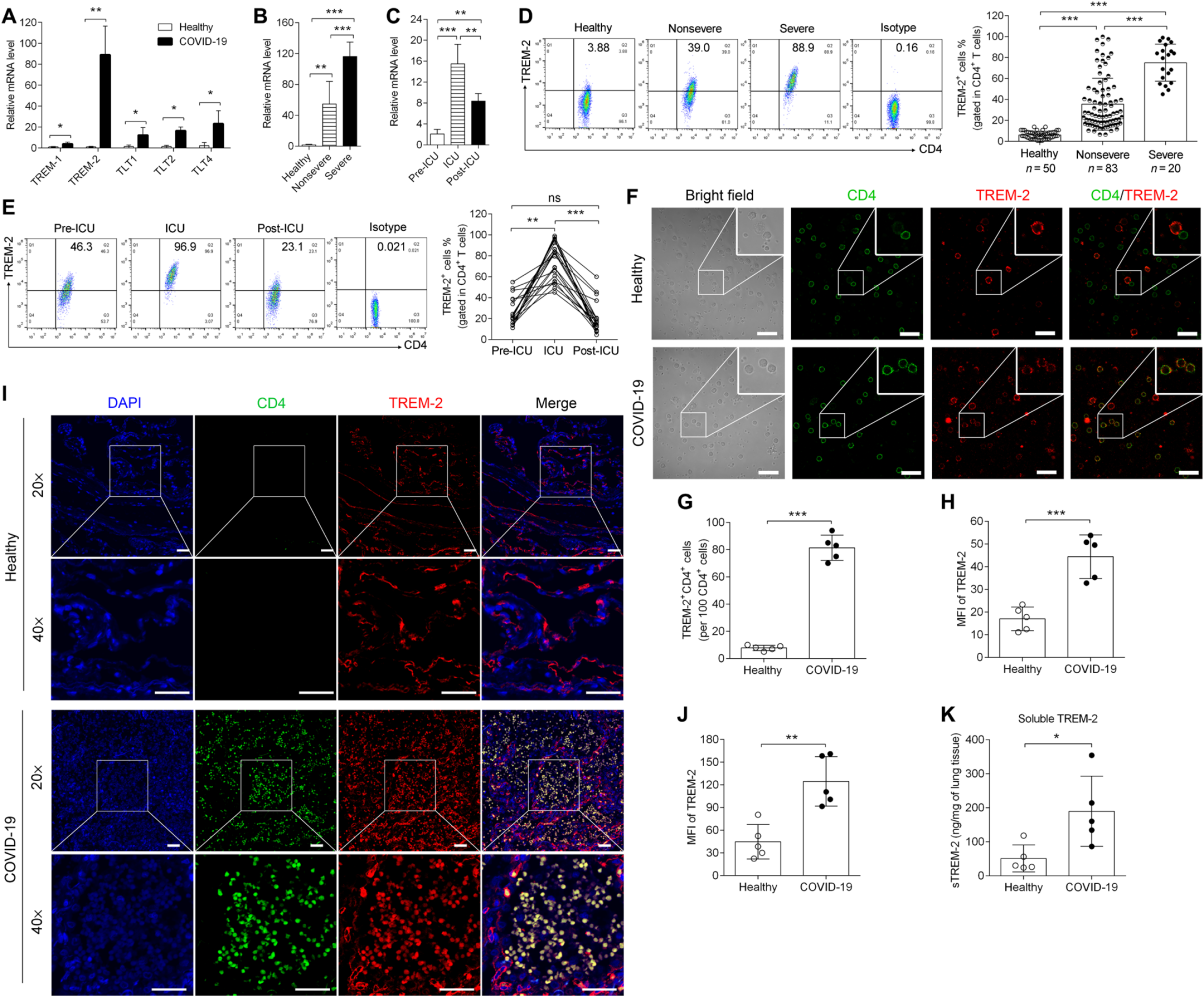
TREM-2 expression on CD4^+^ T cells was increased in patients with COVID-19. (**A**) The expression levels of TREM-1, TREM-2, TLT1, TLT2, and TLT4 in PBMCs from healthy donors or patients with COVID-19 were analyzed by real-time polymerase chain reaction (PCR). (**B** and **C**) The mRNA level of TREM-2 was determined in patients with nonsevere versus severe COVID-19 and patients with severe COVID-19 at the stage of pre-ICU, ICU, and post-ICU. (**D**) Flow cytometric analysis of TREM-2 expression in CD4^+^ T cells from healthy donors (*n* = 50) or patients with nonsevere (*n* = 83) and severe COVID-19 (*n* = 20). (**E**) Flow cytometric analysis of TREM-2 expression in CD4^+^ T cells from patients with severe COVID-19 (*n* = 20) at the stage of pre-ICU, ICU, and post-ICU. (**F**) PBMCs from healthy donors or patients with COVID-19 were double-stained with anti-CD4 (green) and anti–TREM-2 (red) Ab and then observed by fluorescent confocal microscopy. Scale bars, 10 μm. (**G**) The number of TREM-2^+^CD4^+^ cells (yellow) was quantified in CD4^+^ cells (green) from patients with COVID-19 (*n* = 5). (**H**) The MFI of TREM-2 in PBMCs was quantified with ImageJ software (*n* = 5). (**I**) Immunofluorescence analysis of normal lung tissue (defined as healthy) versus pathological lung sections from one critical patient with COVID-19 who died from COVID-19. [4′,6-diamidino-2-phenylindole (DAPI), blue; CD4, green; TREM-2, red]. Scale bars, 50 μm. (**J**) The MFI of TREM-2 was quantified with ImageJ software in five fields of the lung. (**K**) The sTREM-2 was determined by ELISA in the supernatant of lung tissues from normal lung tissues (defined as healthy) versus lung tissues from a critical patient with COVID-19 (*n* = 5). ns, not significant. *P < 0.05, **P < 0.01, and ***P < 0.001.

To explore the expression profile of TREMs during SARS-CoV-2 infection, PBMCs were isolated from the blood of patients with COVID-19 (*n* = 103; table S1) or healthy donors (*n* = 50; table S1). TREM-2 mRNA expression was substantially up-regulated in the PBMCs of patients with COVID-19 compared with the healthy group, while expression of TREM-1, TLT1, TLT2, and TLT4 was slightly increased (Fig. 1A). Furthermore, TREM-2 mRNA expression on PBMCs was analyzed in patients with nonsevere and severe COVID-19, as well as patients admitted to the intensive care unit (ICU). TREM-2 mRNA expression was increased on PBMCs from patients with COVID-19 compared to healthy donors, with the highest abundance in patients with severe COVID-19 (Fig. 1B). Among patients with severe COVID-19, TREM-2 mRNA expression was highly induced when patients were under critical conditions (in ICU) compared to when before and after ICU care (pre-ICU and post-ICU) (Fig. 1C). Next, flow cytometry was used to analyze the surface expression of TREM-2 in subpopulations including monocytes and T cells (fig. S1). Results showed that TREM-2 was equally expressed on CD14^+^ monocytes from nonsevere, patients with severe COVID-19, and healthy controls (fig. S2A). On the contrary, TREM-2 expression was hardly detected on CD4^+^ and CD8^+^ T cells of healthy volunteers, but its expression was highly induced on CD4^+^ and CD8^+^ T cells of patients with COVID-19 (Fig. 1D and fig. S2B). The percentage of TREM-2^+^CD4^+^ T cells was significantly increased in patients with nonsevere (35.56 ± 2.74%) or severe (75.18 ± 3.96%) COVID-19, compared to the low level in healthy donors (6.16 ± 0.49%) (Fig. 1D). The surface TREM-2 was significantly induced on CD4^+^ T cells of patients with ICU period (Fig. 1E). In addition, the percentage of TREM-2^+^CD8^+^ T cells was also up-regulated in patients with nonsevere (18.83 ± 2.15%) or severe (48.9 ± 6.31%) COVID-19, compared with healthy donors (7.2 ± 0.84%) (fig. S2B). As the expression of TREM-2 on CD4^+^ T cells of patients significantly increased, we then focused on the role of TREM-2–expressing CD4^+^ T cells in COVID-19 in the following experiments. Immunofluorescence microscopy detected the localization of TREM-2 on the surface of peripheral CD4^+^ T cell from patients with COVID-19, while no colocalization of TREM-2 with CD4 was observed in healthy controls, consistent with the extremely low level of TREM-2 on CD4^+^ T cells from healthy volunteers (Fig. 1, F and G). The mean fluorescence intensity (MFI) of TREM-2 in PBMCs was significantly higher than these in healthy PBMCs (Fig. 1H). Furthermore, lung tissue collected from patients with COVID-19 (table S2) revealed inflammatory infiltration and destruction compared to healthy donors (fig. S3). We observed that TREM-2 was predominantly expressed in infiltrated CD4^+^ T cells that had infiltrated the lungs of patients with COVID-19, while in the lungs of healthy controls, TREM-2^+^CD4^+ ^T cells were almost undetectable, and TREM-2 was mainly expressed in other types of cells, hypothesized to be alveolar macrophages and epithelial cells (Fig. 1I). In addition, the MFI of TREM-2 in COVID-19 lung was significantly higher than these in healthy lung (Fig. 1J). To investigate the role of TCR signal in TREM-2 expression, CD3 agonist antibody (Ab) was used to stimulate CD4^+^ T cell, and TREM-2 expression was detected. We found that TCR activation did not induce TREM-2 expression in CD4^+^ T cells from healthy donors (fig. S4A). However, plasma from patients with COVID-19 significantly increased TREM-2 expression in healthy CD4^+^ T cells (fig. S4B), which indicated that SARS-CoV-2 and viral induced inflammatory condition were required for TREM-2 expression in T cells. A soluble form of TREM-2 (sTREM-2) is derived from the proteolytic cleavage of the cell surface receptor and is increased in HIV or Sendai virus infection (*27*, *30*). Next, sTREM-2 was detected in the supernatant of lung tissues from patients with COVID-19 and healthy controls. Enzyme-linked immunosorbent assay (ELISA) revealed that sTREM-2 was significantly increased in COVID-19 lungs compared to those in healthy lungs (Fig. 1K). Together, these results indicated a potential role of TREM-2 in the CD4^+^ T cell responses in patients with COVID-19.

To further explore the contribution of TREM-2 in COVID-19, we analyzed the correlations of TREM-2 with disease indexes including C-reactive protein (CRP), lymphocyte count, and D-dimer, as well as age, which are considered as a risk factor in COVID-19 (table S1) (*1*). The frequency of TREM-2^+^CD4^+^ T cells was positively correlated with CRP, D-dimer, and age and negatively correlated with absolute lymphocyte count, which has been significantly reduced in patients with COVID-19 (fig. S5).

T cells display a highly activated phenotype during the acute phase of COVID-19 (*31*). We next investigated the activation phenotype including CD134, CD137, CD69, and CD25 on TREM-2^+^ versus TREM-2^−^CD4^+^ T cells. In patients with COVID-19, TREM-2^+^CD4^+^ T cells showed higher levels of all above activation markers, compared to TREM-2^−^CD4^+^ T cells (fig. S6, A to D). Furthermore, we analyzed the memory phenotype of TREM-2^+^CD4^+^ T cells by flow cytometry with double staining of CD45RO/CCR7 (*12*), including naive T cells (CD45RO^−^CCR7^+^), central memory T cells (T_CM_; CD45RO^+^CCR7^+^), effector memory T cells (T_EM_; CD45RO^+^CCR7^−^), and terminally differentiated effector T cells (T_EMRA_; CD45RO^−^CCR7^−^). The percentages of T_CM_ and T_EM_ cells were increased, while naive T and T_EMRA_ cells were decreased, in TREM-2^+^ versus TREM-2^−^ T cells from patients with COVID-19 (fig. S6E). These data suggested that TREM-2–expressed T cells from patients with COVID-19 exhibited activation and effector memory phenotype.

### TREM-2 bound to SARS-CoV-2 M protein and interacted with CD3ζ/ZAP70 in T cells of patients with COVID-19

**Fig. 2.**
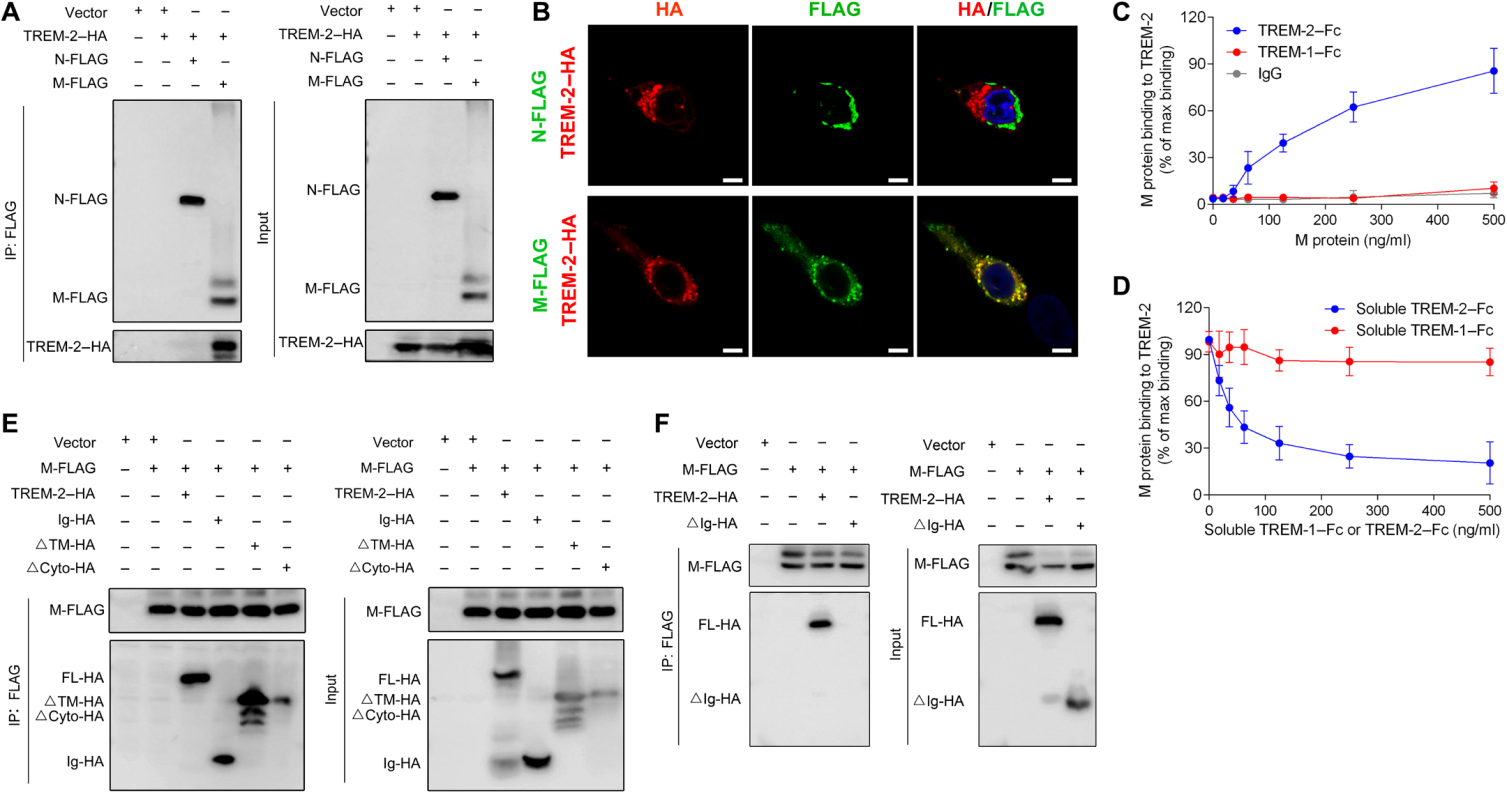
TREM-2 bound SARS-CoV-2 M protein. (**A** and **B**) HEK293T cells were transfected with plasmids containing HA-tagged TREM-2, FLAG-tagged SARS-CoV-2 M protein, and FLAG-tagged SARS-CoV-2 N protein. The interaction of TREM-2 with SARS-CoV-2 M or N protein was analyzed by co-IP assay (A) and confocal scanning microscopy (B) (DAPI, blue; SARS-CoV-2 M, green; TREM-2, red). Scale bars, 10 μm. (**C**) Plates coated with control IgG with same Fc, TREM-1–Fc, or TREM-2–Fc were incubated with increasing concentrations of recombinant M protein, and bound M protein was detected using M protein Ab. (**D**) Plates coated with TREM-2–Fc were incubated with recombinant M protein in the presence of increasing concentrations of TREM-2–Fc or TREM-1–Fc, and bound M protein was detected using M protein Ab. The amount of M protein bound to TREM-2 was then quantified by normalizing the signal intensities of the TREM-2–M protein in which anti–M protein Ab was coated onto the plate and detected by another anti–M protein Ab. (**E** and **F**) HEK293T cells were transfected with plasmids encoding TREM-2 full-length (FL), Ig domain, or truncated forms of TREM-2 respectively deleting transmembrane (ΔTM), cytosolic domain (ΔCyto), or Ig domain (ΔIg). The interaction of TREM-2 and M protein was immunoprecipitated with anti-HA or FLAG Ab and determined with their Ab.

**Fig. 3.**
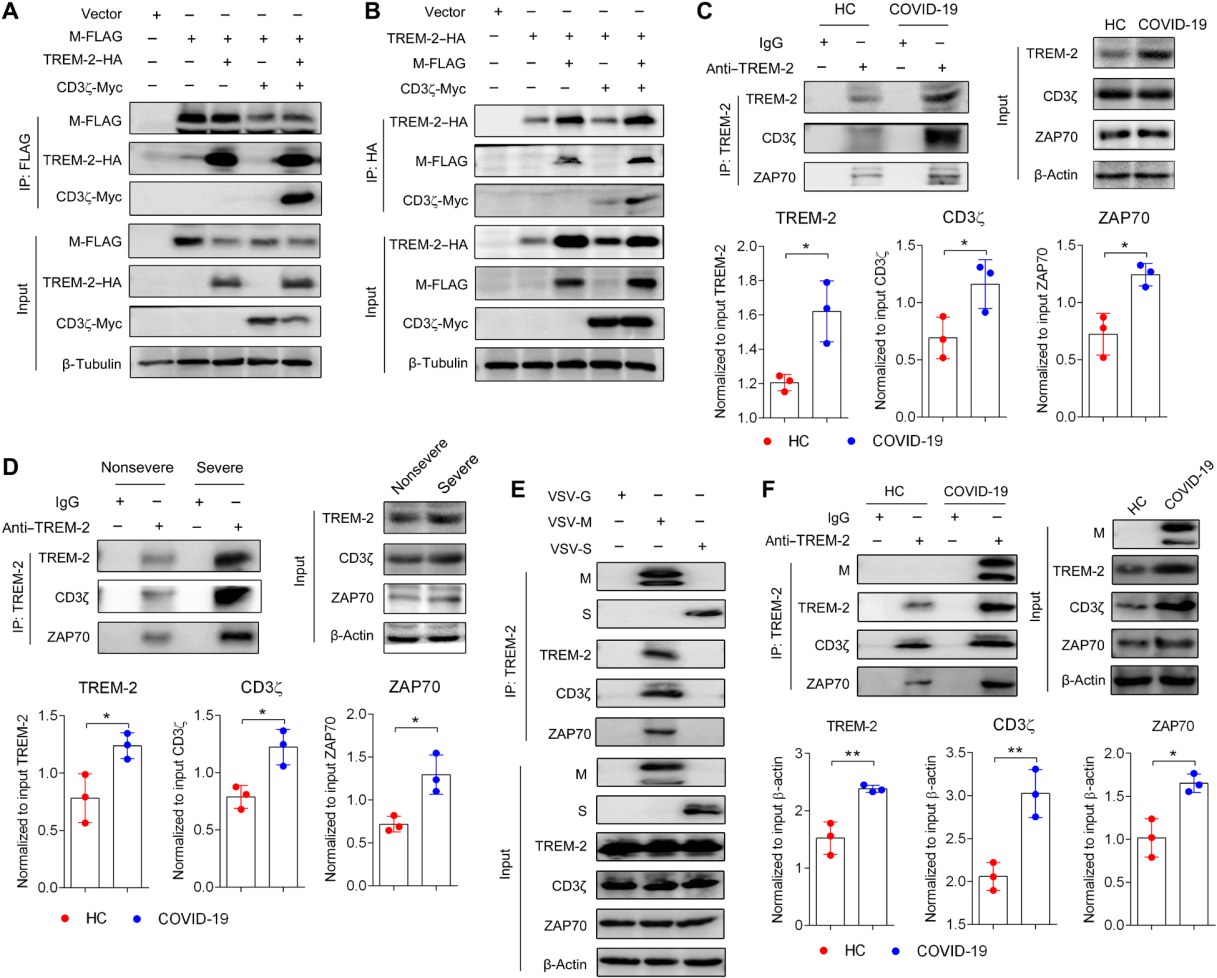
TREM-2 bound SARS-CoV-2 M protein and interacted with CD3ζ/ZAP70. (**A** and **B**) HEK293T cells were transfected with plasmids containing HA-tagged TREM-2, FLAG-tagged SARS-CoV-2 M protein, and Myc-tagged CD3ζ. The interaction of TREM-2, SARS-CoV-2 M protein, and CD3ζ was performed by co-IP assay. Blots of cell lysates (input) or anti-FLAG. (**C** and **D**) Co-IP assay was performed to analyze the interaction among TREM-2, CD3ζ, and ZAP70 in CD4^+^ T cells isolated from healthy donors versus patients with COVID-19, as well as patients with nonsevere versus severe COVID-19. The intensity of anti–TREM-2 IP bands was quantified and normalized to input bands. (**E**) Sorted CD4^+^ T cells from patients with COVID-19 were incubated with pseudovirus reconstituted with SARS-CoV-2 M protein (VSV-M) or S protein (VSV-S). The interaction of TREM-2, ZAP70, CD3ζ, S protein, or M protein was immunoprecipitated with anti–TREM-2 Ab and was determined with their Ab. (**F**) Lung tissues from healthy donor versus patient with COVID-19 were dissociated and immunoprecipitated with anti–TREM-2 Ab. The interaction of TREM-2, ZAP70, CD3ζ, and M protein was determined with their Ab. The intensity of anti–TREM-2 IP bands was quantified and normalized to input β-actin. *P < 0.05 and **P < 0.01.

As a PRR, TREM-2 could recognize the components of pathogens (*32*, *33*), which led us to investigate the possibility that TREM-2 may bind and recognize SARS-COV-2. Immunoprecipitation (IP) was applied to explore the interactions between TREM-2 and SARS-CoV-2 structural proteins including SARS-CoV-2 S, M, and N in human embryonic kidney (HEK) 293T cells. Results identified that TREM-2 could bind to SARS-CoV-2 M protein (Fig. 2A), but not N protein or the receptor binding domain (RBD) of S protein (fig. S7). M but not N protein of SARS-CoV-2 colocalized with TREM-2 in HEK293T cells (Fig. 2B). To confirm the binding of M protein and TREM-2, solid-phase binding assay (*34*) was used to determine the binding of recombinant M protein and TREM-2–Fc. We found that the M protein bound to TREM-2–Fc but not to immunoglobulin G (IgG) or TREM-1–Fc (Fig. 2C). sTREM-2–Fc but not TREM-1–Fc reduced binding of M protein and plate coated with TREM-2–Fc (Fig. 2D). Various lipids and lipoproteins have been reported as the potential ligands for TREM-2 (*34*, *35*). Meanwhile, TREM-2 could recognize the phosphatidylserine (PS) or sphingomyelin (SM) exposed on the cell membrane and promote the phagocytosis of apoptotic cells (*36*). To investigate the role of lipid in M protein–TREM-2 interaction, M protein combined with PS or SM was incubated in plate coated with TREM-2–Fc. We found that PS or SM did not affect the binding of TREM-2 and M protein (fig. S8), which indicated that TREM-2 may interact with M protein independent of viral lipids, at least PS and SM. Furthermore, IP results suggested that Ig domain alone, lacking transmembrane (∆TM) or cytosolic (∆Cyto) forms of TREM-2, could all interact with M protein (Fig. 2E), but this binding disappeared when TREM-2 lacked Ig domain (∆Ig) (Fig. 2F), which indicated that TREM-2 coupled with SARS-CoV-2 M protein by its extracellular Ig domain. To examine the downstream adaptor interacting with TREM-2 in T cell, IP and liquid chromatography–mass spectrometry (LC-MS) were performed in CD4^+^ T cells of patients with COVID-19. Mass spectrum identified an immunoreceptor tyrosine-based activation motif (ITAM) adaptor CD3ζ (CD247), which is a component of TCR complex to assist with T cell antigen recognition (*37*), as a potential protein that interacted with TREM-2 in patients with COVID-19 T cells (table S3). Phosphorylation of CD3ζ ITAMs renders the CD3 chain capable of binding the ZAP70, a protein tyrosine kinase that is essential for T cell activation (*38*). Mass spectra also indicated the interaction of ZAP70 with TREM-2 (table S3). IP data immunoprecipitated with anti-FLAG beads revealed that M protein interacted with TREM-2 alone or with TREM-2/CD3ζ complex, but not CD3ζ alone (Fig. 3A). In addition, IP results with anti-HA beads observed that TREM-2 interacted with CD3ζ or M protein/CD3ζ complex (Fig. 3B). Moreover, we found that the binding of TREM-2 and CD3ζ/ZAP70 complex was enhanced in T cells of patients with COVID-19 versus healthy controls (Fig. 3C) and increased in T cells from severe versus nonsevere patients (Fig. 3D). To confirm that M protein could bind to TREM-2 and interact with CD3ζ/ZAP70 complex in CD4^+^ T cells, sorted CD4^+^ T cells from patients with COVID-19 were treated with pseudovirus reconstituted with M protein or S protein [vesicular stomatitis virus (VSV)–S]. IP data showed that M protein but not S protein interacted with TREM-2/CD3ζ/ZAP70 complex in primary T cell (Fig. 3E). Furthermore, the binding of M protein and TREM-2/CD3ζ/ZAP70 complex was confirmed by IP in COVID-19 lung, which was accumulated with abundant TREM-2^+^CD4^+^ T cells (Fig. 1I). We found that M protein bound to TREM-2/CD3ζ/ZAP70 complex, and this binding was increased in the lung of patients with COVID-19 versus healthy controls (Fig. 3F). Together, these data suggested that TREM-2 bound to SARS-CoV-2 M protein and interacted with CD3ζ/ZAP70 complex in T cells during COVID-19.

### SARS-CoV-2 M/TREM-2 binding induced CD3ζ/ZAP70/STAT1 signaling in T cells

**Fig. 4.**
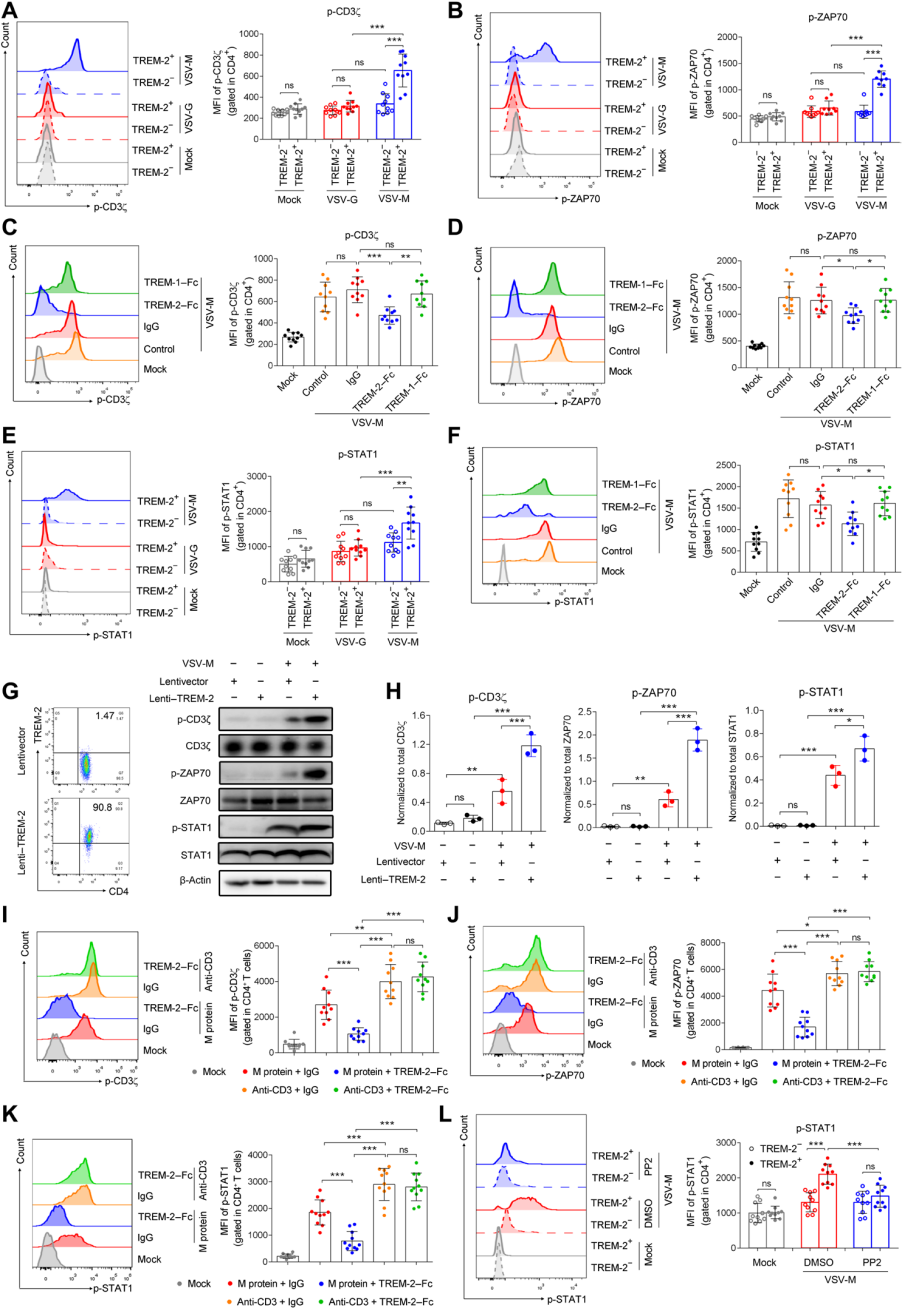
TREM-2 enhanced CD3ζ/ZAP70/STAT1 signal pathway in SARS-CoV-2 M protein–stimulated CD4^+^ T cell. (**A** to **F**) Sorted CD4^+^ T cells from patients with COVID-19 (*n* = 10) were cultured and stimulated with pseudovirus of SARS-CoV-2 M protein for 12 hours, and no stimulation (Mock) and empty pseudovirus vector (Vector) stimulation were used as controls (A, B, and E). Sorted CD4^+^ T cells from patients with COVID-19 (*n* = 10) were cultured and stimulated with pseudovirus of SARS-CoV-2 M protein in the presence of TREM-2–Fc or TREM-1–Fc fusion protein or isotype IgG for 12 hours, and no stimulation (Mock) and only pseudovirus of SARS-CoV-2 M protein stimulation (Control) were used as control (C, D, and F). MFI of phosphorylated CD3ζ (p-CD3ζ) (A and C), ZAP70 (p-ZAP70) (B and D), and STAT1 (p-STAT1) (E and F) in TREM-2^−^ versus TREM-2^+^CD4^+^ T cells were analyzed by flow cytometry. (**G**) Purified CD4^+^ T cells were treated with lentivirus containing TREM-2 and then stimulated with pseudovirus of SARS-CoV-2 M protein. Flow cytometric analysis of surface TREM-2 in CD4^+^ T cells overexpressed TREM-2. p-CD3ζ, p-ZAP70, and p-STAT1 were analyzed by Western blot. (**H**) The intensity of phosphorylated bands in (G) was quantified and normalized to total bands. (**I** to **K**) Sorted CD4^+^ T cells from patients with COVID-19 (*n* = 10) were cultured and stimulated with recombinant M protein or anti-CD3 Ab in the presence of TREM-2–Fc fusion protein or isotype IgG for 12 hours. MFI of p-CD3ζ (I), p-ZAP70 (J), and p-STAT1 (K) in CD4^+^ T cells were analyzed by flow cytometry. (**L**) Sorted CD4^+^ T cells from patients with COVID-19 were stimulated with pseudovirus of SARS-CoV-2 M protein in the presence of ZAP-70 inhibitor PP2 or dimethyl sulfoxide (DMSO). MFI of p-STAT1 in TREM-2^−^ versus TREM-2^+^CD4^+^ T cells were analyzed by flow cytometry. *P < 0.05, **P < 0.01, and ***P < 0.001.

We next assessed the signal transduction mediated by TREM-2 in T cells from patients with COVID-19. Using an infectious molecular clone of VSV system, we replaced the glycoprotein gene (VSV-G) with the M protein of SARS-CoV-2 (VSV-M) and developed a pseudovirus reconstituted with M protein (*39*, *40*). Higher levels of phosphorylated CD3ζ (Tyr^83^) (Fig. 4A) and ZAP70 (Tyr^319^) (Fig. 4B) were detected in pseudovirus–M protein–stimulated TREM-2^+^CD4^+^ T cells from patients with COVID-19 compared with TREM-2^−^ groups, whereas pseudovirus vector did not induce the phosphorylations of CD3ζ and ZAP70 in TREM-2^+^CD4^+^T cells (Fig. 4, A and B). TREM-2–Fc fusion protein, used to block the interaction between TREM-2 and M protein, reduced the phosphorylation of CD3ζ (Tyr^83^) and ZAP70 (Tyr^319^) in CD4^+^ T cells of patients with COVID-19 upon pseudovirus–M protein stimulation, whereas control IgG did not affect the phosphorylation of CD3ζ/ZAP70 (Fig. 4, C and D). However, the unrelated fusion protein TREM-1–Fc could not block the activation of CD3ζ/ZAP70 in pseudovirus–M protein–stimulated CD4^+^ T cells (Fig. 4, C and D). TCR stimulation could trigger STAT1 (Ser^727^) phosphorylation in the absence of IFN-γ signaling (*41*). Pseudovirus–M protein also activated STAT1 phosphorylation (Ser^727^) in TREM-2^+^CD4^+^ T cells (Fig. 4E). As expected, TREM-2–Fc but not TREM-1–Fc fusion protein suppressed the phosphorylation of STAT1 (Ser^727^) induced by pseudovirus–M protein (Fig. 4F). In addition, overexpression of TREM-2 increased CD3ζ (Tyr^83^), ZAP70 (Tyr^319^), and STAT1 (Ser^727^) phosphorylation upon pseudovirus–M protein stimulation (Fig. 4, G and H).

To confirm the role of M protein/TREM-2 binding in activation of CD3ζ/ZAP70/STAT1 signal pathway, recombinant M protein was used as a stimulator, and anti-CD3 agonist Ab was used as a positive control in T cell activation. Both M protein and anti-CD3 Ab induced CD3ζ (Tyr^83^), ZAP70 (Tyr^319^), and STAT1 (Ser^727^) phosphorylation in COVID-19 CD4^+^ T cells (Fig. 4, I to K). TREM-2–Fc significantly reduced the phosphorylation of CD3ζ (Tyr^83^), ZAP70 (Tyr^319^), and STAT1 (Ser^727^) induced by M protein but not those induced by anti-CD3 Ab (Fig. 4, I to K). Next, we used TREM-2 Ab to block M protein/TREM-2 binding and observed that TREM-2 Ab inhibited the phosphorylation of CD3ζ (Tyr^83^), ZAP70 (Tyr^319^), and STAT1 (Ser^727^) induced by M protein in CD4^+^ T cells (fig. S9). Furthermore, we observed that the phosphorylation of STAT1 was enhanced in human TREM-2^+^ versus TREM-2^−^CD4^+^ T cells upon stimulation with pseudovirus–M protein, but this effect was blocked by ZAP70 inhibitor PP2 (Fig. 4L). Moreover, TREM-2 also induced activation of CD3ζ/ZAP70/STAT1 signal pathway in CD8^+^ T cells from patients with COVID-19 (fig. S10). To further exclude the role of viral lipid in activation of TREM-2/CD3ζ/ZAP70/STAT1 signal pathway mediated by M protein, PS/SM alone or combined with M protein was used to stimulate T cells. We found that PS or SM alone did not induce the phosphorylation of CD3ζ (Tyr^83^), ZAP70 (Tyr^319^), and STAT1 (Ser^727^), and they combined with M protein did not enhance M protein–mediated CD3ζ/ZAP70/STAT1 signal activation (fig. S11). These results together suggested that M protein–TREM-2 interaction activated CD3ζ/ZAP70/STAT1 signaling axis in T cells during SARS-CoV-2 infection.

STAT1 forms homodimers or heterodimers to induce T_H_1 differentiation, which is featured by enhanced T-bet transcription (*42*). Next, we analyzed T-bet expression in TREM-2^+^ and TREM-2^−^CD4^+^ T cells of patients with COVID-19 by flow cytometry and detected up-regulated T-bet expression in TREM-2^+^ versus TREM-2^−^CD4^+^ T cells (fig. S12A). Consistently, blockage of TREM-2 reduced the expression of T-bet induced by pseudovirus–M protein (fig. S12B). In addition, ZAP70 inhibitor PP2 or STAT inhibitor SH-4-54 inhibited the induction of T-bet expression in TREM-2^+^CD4^+^ T cells upon pseudovirus–M protein stimulation (fig. S12C). Furthermore, recombinant M protein and anti-CD3 Ab induced T-bet expression in CD4^+^ T cells (fig. S12D). TREM-2–Fc significantly reduced T-bet expression induced by M protein but not those induced by anti-CD3 Ab (fig. S12D). Collectively, these results suggested that SARS-CoV-2 activated TREM-2–mediated T-bet expression via ZAP70/STAT1 signaling in T cells.

### TREM-2 facilitated T_H_1 cytokine production in T cells from patients with COVID-19

**Fig. 5.**
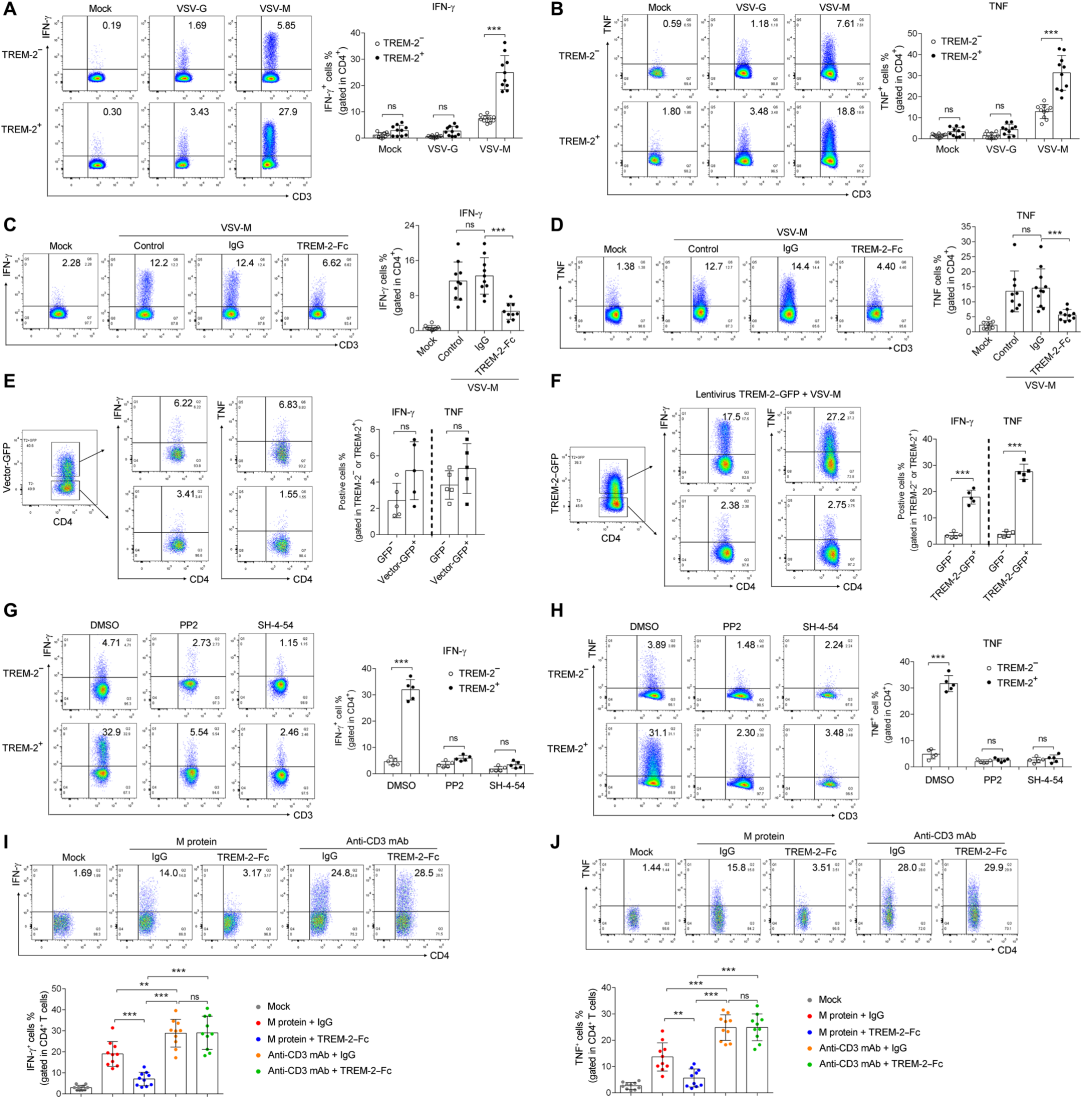
TREM-2 facilitated T_H_1 cytokine production in CD4^+^ T cells in COVID-19. (**A** and **B**) Sorted CD4^+^ T cells from patients with COVID-19 (*n* = 10) were cultured and stimulated with pseudovirus of SARS-CoV-2 M protein for 12 hours, and no stimulation (Mock) and pseudovirus vector (Vector) stimulation were used as controls. (**C** and **D**) Sorted CD4^+^ T cells from patients with COVID-19 (*n* = 10) were cultured and stimulated with pseudovirus of SARS-CoV-2 M protein in the presence of TREM-2–Fc fusion protein or isotype IgG for 12 hours, and no stimulation (Mock) and only pseudovirus of SARS-CoV-2 M protein stimulation (control) were used as controls. (**E** and **F**) Purified CD4^+^ T cells were treated with lentivirus containing TREM-2 (F) or vector (E) and then stimulated with pseudovirus of SARS-CoV-2 M protein. (**G** and **H**) Sorted CD4^+^ T cells from a patient with COVID-19 were stimulated with pseudovirus of SARS-CoV-2 M protein in the presence of ZAP-70 inhibitor PP2, pan-STATs inhibitor SH-4-54 or DMSO. (**I** and **J**). Sorted CD4^+^ T cells from a patient with COVID-19 patient were stimulated with recombinant M protein or anti-CD3 Ab in the presence of TREM-2–Fc fusion protein or isotype IgG for 12 hours. Cells were then stimulated with phorbol 12-myristate 13-acetate (50 nM), ionomycin (1 μg/ml), and brefeldin A (1 μg/ml) for 6 hours. Percentages of IFN-γ– and TNF-α–producing cells were analyzed by flow cytometry. ns, not significant; **P < 0.01; ***P < 0.001. mAb, monoclonal Ab.

Because SARS-CoV-2 infection induced proinflammatory T_H_1 responses (*11*–*13*), we next evaluated the role of TREM-2 on modulating T_H_1 cytokines production. Upon stimulation with pseudovirus–M protein but not pseudovirus-vector ex vivo, production of IFN-γ and TNF was increased in TREM-2^+^CD4^+^ and CD8^+^ T cells of patients with COVID-19 (Fig. 5, A and B, and fig. S13, A and B). Consistently, TREM-2–Fc fusion protein reduced IFN-γ and TNF production using CD4^+^ and CD8^+^ T cells of patients with COVID-19 (Fig. 5, C and D, and fig. S13, C and D). CTL-mediated antiviral responses occurred in patients with COVID-19 during SARS-COV-2 infection (*11*, *43*). We also observed that the production of granzyme B, a cytotoxic protease, was elevated in TREM-2^+^CD8^+^ T cells of patients with COVID-19 compared to TREM-2^−^ group after pseudovirus–M protein stimulation ex vivo (fig. S13E), and blockage of TREM-2 signal with TREM-2–Fc fusion protein reduced production of granzyme B by CD8^+^ T cells (fig. S13F). However, TREM-2 did not enhance IL-1β and IL-6 production by CD4^+^ T cell upon pseudovirus–M protein stimulation (fig. S14). To further confirm the intrinsic role of TREM-2 in T_H_1 response, TREM-2 was overexpressed in purified CD4^+^ T cells from healthy donors by lentivirus-packaged TREM-2 with green fluorescent protein (GFP) tag. We observed that GFP^+^ (TREM-2^+^) CD4^+^ T cells produced more IFN-γ and TNF than GFP^−^ (TREM-2^−^) subset upon pseudovirus–M protein stimulation (Fig. 5, E and F). Furthermore, IFN-γ and TNF production induced in TREM-2^+^CD4^+^ T cells were substantially inhibited by treatment of PP2 or SH-4-54 (Fig. 5, G and H). Next, recombinant M protein was used as a stimulator, and anti-CD3 agonist Ab was used as a positive control in T_H_1 cytokine production. We found that both recombinant M protein and anti-CD3 Ab significantly induced IFN-γ and TNF production by CD4^+^ T cell (Fig. 5, I and J). TREM-2–Fc significantly reduced the IFN-γ and TNF production induced by M protein but not those induced by anti-CD3 Ab (Fig. 5, I and J). Furthermore, TREM-2 Ab treatment, blocking M protein/TREM-2 binding, significantly reduced IFN-γ and TNF production induced M protein (fig. S15). To further exclude the role of viral lipid in IFN-γ and TNF production, recombinant M protein was used to stimulate CD4^+^ T cell. Lipid component PS or SM could not induce IFN-γ and TNF production (fig. S16) and did not enhance IFN-γ and TNF production in CD4^+^ T cells induced by M protein (fig. S16). Together, our data suggested that TREM-2 promoted T_H_1 and cytotoxic responses of T cells in patients with COVID-19.

### TREM-2 enhanced T_H_1 response and protection in coronavirus infection model

**Fig. 6.**
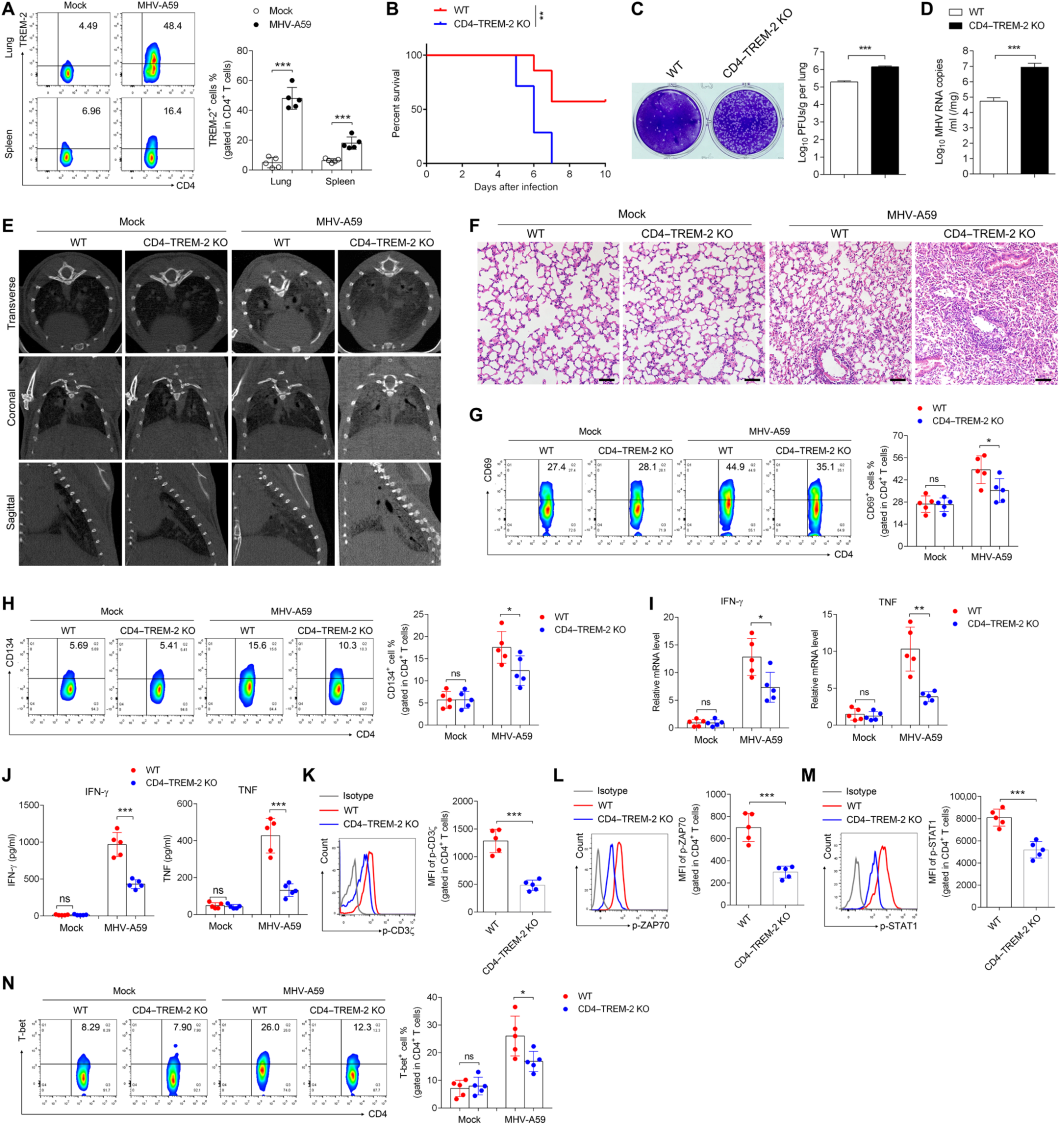
TREM-2 promoted T_H_1-mediated host defense against coronavirus infection. (**A**) Flow cytometric analysis of TREM-2 expression on CD4^+^ T cells from the lungs and spleen of MHV-A59–infected WT mice versus MHV-A59–infected CD4–TREM-2 KO mice. (**B**) Survival rate of WT mice versus CD4–TREM-2 KO mice infected with MHV-A59. (**C** and **D**) The expression levels of MHV N gene (C) and viral load (D) in lungs of infected mice were analyzed by real-time PCR and plaque assay. (**E**) CT imaging of uninfected mice and WT mice versus CD4–TREM-2 KO mice. (**F**) Hematoxylin and eosin staining of lung sections was examined in uninfected mice and WT mice versus CD4–TREM-2 KO mice. (**G** and **H**) The expression of CD69 (G) and CD134 (H) was determined in lung-infiltrating CD4^+^ T cells by flow cytometry. (**I** and **J**) The mRNA expression in the lung (I) and protein level in the plasma (J) for IFN-γ and TNF-α were analyzed in MHV-A59–infected WT mice versus CD4–TREM-2 KO mice by real-time PCR and ELISA. (**K** to **N**) MFI of p-CD3ζ (K), p-ZAP70 (L), and p-STAT1 (M) as well as T-bet expression (N) in WT versus CD4–TREM-2 KO CD4^+^ T cells was analyzed by flow cytometry. The uninfected mice were as control (Mock). Scale bars, 50 μm. *P < 0.05, **P < 0.01, and ***P < 0.001.

SARS-CoV-2 and murine coronavirus MHV-A59 induce similar immune responses in mouse model (*44*). To examine whether TREM-2 influences CD4^+^ T cell–mediated anti-coronavirus responses, WT and TREM-2–CD4 conditional KO (cKO) (CD4–TREM-2 KO) mice were intranasally inoculated with MHV-A59 according to previous studies (*44*, *45*). Results showed an increased frequency of TREM-2^+^CD4^+^ T cells in the lungs and spleens of C57BL/6 mice after MHV-A59 infection (Fig. 6A). CD4–TREM-2 KO mice displayed reduced survival ratio after MHV-A59 infection (Fig. 6B). Furthermore, the virus load of MHV-A59 was significantly increased in the lung of CD4–TREM-2 KO mice (Fig. 6, C and D), suggesting the impaired ability of MHV-A59 clearance. Furthermore, computed tomography (CT) morphology revealed that high density increased throughout the lungs (typical signs of acute pneumonia) after MHV-A59 infection in CD4–TREM-2 KO versus WT mice after 5 days intranasally (Fig. 6E), and hematoxylin and eosin staining showed that the deficiency of TREM-2 in CD4^+^ T cells accelerated the lung destruction (Fig. 6F). Furthermore, we found that deficiency of TREM-2 reduced the activation marker CD69 and CD134 in the lung-infiltrating CD4^+^ T cell (Fig. 6, G and H) and CD8^+^ T cells (fig. S17). Meanwhile, a decrease of IFN-γ and TNF was observed in the lung (Fig. 6I) and serum (Fig. 6H) of TREM-2–CD4 cKO versus WT mice. The downstream signaling molecules that interacted with TREM-2, as we observed in patients with COVID-19, including *CD247* (CD3ζ), *ZAP70*, *STAT1*, and *TBX21* (T-bet), were reduced in the lung of TREM-2–CD4 cKO mice compared to WT mice (fig. S18). In addition, we found that phosphorylation of CD3ζ (Tyr^83^) (Fig. 6K), ZAP70 (Tyr^319^) (Fig. 6L), and STAT1 (Ser^727^) (Fig. 6M), as well as the expression of T-bet (Fig. 6N), was reduced in lung CD4^+^ T cells of CD4–TREM-2 KO mice upon MHV-A59 infection. These results together suggested that TREM-2 in CD4^+^ T cells promoted T_H_1 differentiation to defend against coronavirus infection in vivo.

## DISCUSSION

In this study, we demonstrated that TREM-2 was inducibly expressed on T cells in response to SARS-CoV-2 infection and interacted with SARS-CoV-2 M protein to activate CD3ζ/ZAP70/STAT1 signaling and proinflammatory T_H_1 responses. We used a TREM-2 cKO mouse model of MHV-A59 infection to show that intrinsic TREM-2 in T cell promoted viral clearance and alleviated the destruction of lung. To the best of our knowledge, this study explored a novel role of TREM-2 in the T cell response, which may explain the inflammatory syndrome and previous in vivo and in vitro studies on SARS-CoV-2 infection.

TREM family was originally identified as innate receptors expressed on myeloid lineage. To date, TLT2 is reported being expressed on B lymphoid lineage cells (*46*), CD8^+^ T cells, and activated CD4^+^ T cells (*25*). We previously reported that TREM-1 was expressed on innate lymphocyte Vδ2T cells and activated the antigen presentation activity of Vδ2T cells to enhance T_H_1 response in TB (*24*). In addition, TREM-2 expression was reported to be induced on peripheral blood CD4^+^ and CD8^+^ T cells in patients with TB (*26*). In this study, we demonstrated that TREM-2 was inducibly expressed on T cells in response to SARS-CoV-2 infection and substantially elevated in patients with severe COVID-19. Furthermore, we found that anti-CD3 agonist Abs could not induce TREM-2 up-regulation in T cells, which indicated that TREM-2 expression was increased in COVID-19 T cells independent of TCR stimulation. However, TREM-2 expression was up-regulated in T cells upon the treatment of plasma from patients with COVID-19, which together indicate that some plasma components such as inflammatory cytokines may induce TREM-2 expression in patients with COVID-19. Meanwhile, TREM-2 expression has been reported to be altered in the in vitro stimulation of inflammatory contexts, including IFN-γ, TNF, IL-1β, and IL-4 (*47*), which have been found in patients with COVID-19, especially in severe patients (*48*). Moreover, the frequency of TREM-2^+^CD4^+^ T cell was positively correlated with CRP, lymphocyte count, and D-dimer, the indicators of patients with severe COVID-19 (*1*). An sTREM-2 is derived from the proteolytic cleavage of the cell surface receptor (*49*). sTREM-2 was elevated in the lung of Sendai virus–infected mice (*27*) or in the cerebrospinal fluids of patients with HIV (*30*). In the study, we found that the concentration of sTREM-2 was increased in the lung tissues from patients with COVID-19. This finding indicates that TREM-2 may be used as a potential biomarker for COVID-19 severity.

The balance between the resting and activation status, naive, and memory phenotype of T cells is crucial for maintaining an efficient immune response (*50*). The study has demonstrated that high expression of CD69, CD134, and CD137 was distinguished as SARS-CoV-2–specific T cells, which produced high-level IFN-γ and granzyme B (*11*). Other study showed that an increase in coexpression of activation phenotype CD38 and human leukocyte antigen DR (HLA-DR) was observed in T cells of patients with COVID-19 (*51*). In the present study, we found that TREM-2 expression on CD4^+^ and CD8^+^ T cells was positively associated with the expression of TCR-dependent activation-induced markers, including CD134 (OX40), CD137 (4-1BB), CD69, and CD25. Increased frequency of effector and memory population was observed in the peripheral T cell of a patient with COVID-19 (*12*). Moreover, we found that TREM-2 expression level on naive CD4^+^ and CD8^+^ T cells was much lower than that in effector and memory T cell subsets (T_CM_ and T_EM_). These data together suggest that TREM-2 may play an essential role in T cell activation and differentiation during COVID-19.

To date, the ligands of TREM-2 are still unclear. It has been reported previously that TREM-2 can bind to certain bacterial components (*32*, *33*) and endogenous proteins (*52*–*54*), especially lipoprotein (*34*, *55*, *56*). The coronavirus M protein is the most abundant structural protein anchoring on the envelope membrane surface of coronavirus and attributes to virion invasion and assembly (*57*). Besides, coronavirus M protein has been proven to play critical roles in host immune responses, including the inducement of Abs (*58*), IFN-α production (*59*), and CTL-specific responses (*60*). A study has demonstrated that the viral epitopes from SARS-CoV-2 M protein could elicit higher percentage of IFN-γ–producing T cells (59%), compared to the S protein (26%) and N protein (22%) peptides (*61*), indicating the predominance of M protein in host T cell responses against SARS-CoV-2. However, because of the lack of related researches, the underlying mechanism associated with T cell activation by SARS-CoV-2 M protein remains unclear. PRRs on T cells could sense the PAMPs and initiate the activation of T cells. SARS-CoV-1 M protein is already identified as a PAMP to trigger TLR-mediated signal (*62*). Here, we identified SARS-CoV-2 M protein as a novel exogenous ligand of TREM-2 by immunofluorescence, co-IP, and in vitro binding assay both in 293T cell, primary T cells, and the lung tissue of patients with COVID-19.

However, it is intriguing that the lymphocytes lack angiotensin-converting enzyme 2 (ACE2) expression (*63*), suggesting an alternative mechanism by which SARS-CoV-2 regulates T lymphocytes. The unique location of M protein on the viral envelope makes it hard to recognize M protein by surface receptors because of the spike structure of S protein. In addition, it is generally believed that the S protein of SARS-CoV-2 is responsible for both virion attachment and internalization (*64*, *65*). However, coronaviruses M protein could mediate the receptor binding and membrane fusion of the virion with the host cell (*66*). For instance, human coronavirus NL63 (HCoV-NL63) uses ACE2 as an entry receptor for infection (*67*). Further study demonstrated that the M protein of NL63 is responsible for this attachment and interaction with adhesion receptor heparan sulfate proteoglycans (*68*). In the study, we could not exclude the possibility that TREM-2 could directly recognize the M protein of SARS-CoV-2 and mediate the attachment of SARS-CoV-2 to the T cells and the subsequent activation of T cell responses. Whereas, other possibilities could also be proposed rationally according to the results we observed. One possibility is the existence of dissociative M protein. In the process of virus assembly, structural and nonstructural proteins are synthesized within target cells. However, some infected cells would be directly lysed by CTLs before the end of virus assembly, which leads to the release of dissociative M protein (*69*). Moreover, the direct target of Abs and complement may also result in the destruction of virus structure and the dissociation of M protein (*70*). Dissociative M protein is recognized and bound with TREM-2, triggering the activation of T cells. This could explain why we detected the binding of M protein with TREM-2 and CD3ζ as well as ZAP70 in the lung tissue of patients with COVID-19, where abundant infiltrating TREM-2^+^CD4^+^ T cells were found. Another possibility is related to the involvement of sTREM-2. sTREM-2 as the secreted form from proteolytic cleavage of the cell surface receptor may represent another binding approach for M protein and TREM-2. sTREM-2 could recognize M protein across the viral envelope and transmit signal. We demonstrated in in vitro binding assay that TREM-2–Fc fusion protein, which is similar to sTREM-2, could bind to recombinant M protein.

Various lipids and lipoproteins have been reported as the potential ligands for TREM-2 (*34*–*36*). It is unclear whether TREM-2 recognizes other components such as lipids, which was expressed in viral membrane, as well as in the sera of patients with severe COVID-19 (*71*). TREM-2 could recognize the lipid component PS or SM exposed on the cell membrane (*36*). PS or SM could not increase the binding between TREM-2–Fc and M protein. Furthermore, PS or SM alone could not induce the activation of CD3ζ/ZAP70/STAT1 signal pathway in T cells. Besides, M protein combined with PS or SM did not enhance the activation of CD3ζ/ZAP70/STAT1 signal pathway compared with the treatment with M protein alone. The results partially demonstrated that M protein bound to TREM-2 and activated TREM-2/CD3ζ/ZAP70/STAT1 signal pathway independent of lipids, at least PS and SM.

TREM-2 was often described as an anti-inflammatory and reparative receptor (*18*, *20*). Nevertheless, TREM-2 signal was reported to be proinflammatory and destructive in vivo in some infectious models, intestinal and neurological disease (*22*, *23*, *72*). Several studies reported that TREM-2 exhibits a distinct role in virus infection in vivo, which indicated that different mechanism participated in TREM-2–mediated viral immunity (*27*–*29*). TREM-2 KO mice showed marked decreases in lung inflammation and airway mucus production after mouse parainfluenza Sendai virus infection (*27*), because TREM-2 allows for macrophages to accumulate in the lung and thereby amplify disease-promoting IL-13 production (*27*). TREM-2 suppresses the proinflammatory response to facilitate porcine reproductive and respiratory syndrome virus infection via PI3K/NF-κB (phosphatidylinositol 3-kinase/nuclear factor κB) signaling (*28*). TREM-2 KO mice were protected from lymphocytic choriomeningitis virus–induced hepatitis and showed improved virus control despite comparable virus-specific T cell responses (*29*). Here, we used CD4-specific conditional TREM-2 KO mice to generate MHV-A59 intranasally inoculated model. CD4-specific conditional TREM-2 KO mice exhibited lower levels of activation markers CD69 and CD134, as well as T_H_1 cytokines IFN-γ and TNF, together with higher viral load and lung destruction after MHV-A59 infection compared with WT mice, suggesting that TREM-2 is required in T cell–mediated immune defense and inflammation.

We explored the signal transduction mechanism of TREM-2–mediated T cell activation. We used IP and LC-MS to find the protein that interacted with TREM-2, and LC-MS data identified that CD3ζ may be the most likely protein. It is well known that antigen-specific signal via TCR induces the phosphorylation of ITAM on CD3 chains and then recruits the kinase ZAP70 to the phosphorylated ITAMs (*37*). Activated ZAP70, in turn, phosphorylates and activates various downstream signal transduction molecules, leading to T cell activation (*38*). In the present study, TREM-2 interacted with CD3ζ/ZAP70 complex in T cells, and this interaction was strengthened in patients with COVID-19 compared to healthy donors. Our data indicated that CD3ζ, rather than DAP12 (the adaptor molecule reported in macrophage) (*73*), may be an adaptor of TREM-2 in T cells. Furthermore, while interacting with SARS-CoV-2 M protein, TREM-2 recruited CD3ζ/ZAP70 complex to activate STAT1/T-bet signaling and promoted proinflammatory T_H_1 responses. Thus, roles of TREM-2 in host inflammation and disease progression may largely depend on the major effector cells belonging to either innate or adaptive immunity.

In summary, the present study explored the role of TREM-2 on T cells in COVID-19. We found that TREM-2 was induced on T cell surface during COVID-19 disease and was bound to SARS-CoV-2 M protein. TREM-2 interacted with CD3ζ/ZAP70 complex in T cells and therefore activated STAT1/T-bet signaling to enhance the proinflammatory T_H_1 responses. These findings have broadened the TREM-2–mediated T cell response of COVID-19 on a new and unexpected mechanism for the regulation of adaptive immunity and host inflammation, which may provide a promising therapeutic target for COVID-19 diseases.

## MATERIALS AND METHODS

### Ethics statement

This study and all experiment protocols were approved by the Ethics Committee Board for Human Experiments in the Fifth Affiliated Hospital of Sun Yat-Sen University (approval number K174-1). For experiments with human samples, informed consent was obtained from all participants. The procedure was performed in accordance with the National Commission for the Protection of Subjects of Biomedical and Behavioral Research guidelines for animal experiments. All efforts were made to minimize suffering.

### Human subjects

Patients with COVID-19 (*n* = 113) were recruited from the Fifth Affiliated Hospital of Sun Yat-sen University (Zhuhai, China). All the patients were selected on the basis of clinical diagnosis and laboratory information. Healthy donors (*n* = 50) were randomly recruited from individuals undergoing health checkup at the Fifth Affiliated Hospital of Sun Yat-sen University and confirmed as coronavirus nucleic acid negative. Detailed clinical characteristics and laboratory information are shown in table S1. Pathological tissues of the lung organ of a patient who died from COVID-19 were provided by the Shenzhen Third People’s Hospital (Shenzhen, China). The lung tissues defined as healthy control were paracancerous normal tissues isolated from the lungs of patients with lung cancer admitted to the Fifth Affiliated Hospital of Sun Yat-sen University. Informed consent was obtained. Detailed clinical characteristics of these patients are shown in table S2.

### Quantitative real-time polymerase chain reaction

Total RNA was extracted from PBMCs using TRIzol (Invitrogen) reagent according to the manufacturer’s protocol, and total RNA yield was quantified using NanoDrop (Thermo Fisher Scientific, Waltham, MA). cDNAs were synthesized from 1 μg of total RNA using the RevertAid First Strand cDNA Synthesis Kit (Thermo Fisher Scientific, Waltham, MA, USA). Quantitative real-time polymerase chain reaction (PCR) was carried out using cDNA, SYBR green PCR master mix (Applied Biosystems, Foster City, CA, USA), and forward and reverse primers for TREM-1, TREM-2, TLT1, TLT2, TLT, IFN-γ, TNF-α, IL-6, IL-1β, and MHV-A59 N gene using a real-time PCR system (CFX96, Bio-Rad Laboratories, Hercules, CA). Relative gene expression was normalized against β-actin, and fold change in mRNA expression was determined using the ΔΔ*C*_t_ method. Primers used in these experiments are shown in table S4.

### Immunofluorescence

PBMCs were centrifuged at 1500 rpm for 5 min and then lysed with 1× red blood cell lysing buffer [BD Biosciences (BD)] for 5 min at room temperature. Cells were fixed with 1% paraformaldehyde (Sigma-Aldrich) in phosphate-buffered saline (PBS) for 15 min and then stained with fluorescein isothiocyanate–labeled anti-CD4 (clone L200, BD) and phycoerythrin (PE)–labeled anti–TREM-2 Abs (clone 237920, R&D Systems) for 30 min. HEK293T cells were fixed for 10 min at room temperature with 4% paraformaldehyde in PBS and permeabilized with 0.1% Triton X100 in 5% bovine serum albumin (BSA) for 1 hour at room temperature. Samples were then incubated with primary Abs to HA [clone C29F4, Cell Signaling Technology (CST)] and FLAG (M2, monoclonal; Sigma-Aldrich) and followed by incubation with Alexa Fluor 488– and Alexa Fluor 594–conjugated anti-rabbit and anti-mouse secondary Abs (Invitrogen). Lung sections from a healthy donor and patient were fixed with 4% paraformaldehyde in PBS and incubated with 5% BSA for 1 hour at room temperature. Samples were then incubated with primary Abs to CD4 (clone RPA-T4, BioLegend) and TREM-2 (polyclonal goat IgG, R&D Systems) at 4°C for 12 hours and followed by incubation with Alexa Fluor 488– and Alexa Fluor 594–conjugated anti-mouse and anti-goat secondary Abs (Invitrogen) for 2 hours. All samples were covered with a drop of 4′,6-diamidino-2-phenylindole (DAPI) for 5 min. Confocal images were captured on a ZEISS LSM 880 confocal microscope using a 20× objective or a 63× oil objective. The quantitative intensity analysis of TREM-2 in confocal images has been performed with ImageJ software.

### Flow cytometry

Fluorescent dye–labeled Abs were purchased from the following companies. Anti-human: CD3 (clone UCHT1, BD), CD4 (clone L200, BD), CD8 (clone RPA-T8, BD), CD14 (clone 63D3, BioLegend), CD45RO (clone UCHL1, BD), CCR7 (clone 150503, BD), CD134 (clone ACT35, BioLegend), CD137 (clone 4B4-1, BioLegend), CD69 (clone FN50, eBioscience), CD25 (clone BC96, eBioscience), TREM-2 (clone 237920, R&D Systems), TNF (clone MAb11, eBioscience), IFN-γ (clone 4S.B3, eBioscience), IL-2 (clone MQ1-17H12, BioLegend), granzyme B (clone QA16A02, BioLegend), T-bet (clone O4-46, BD), anti–phospho-ZAP70 (Tyr^319^) (clone 1503310, BioLegend), anti–phospho-CD3 (Tyr^83^) [clone EP776(2)Y, Abcam], and anti–phospho-STAT1 (Tyr^701^) (clone A15158B, BioLegend). Anti-mouse: CD3 (clone 17A2, BioLegend), CD4 (clone GK1.5, BioLegend), CD8 (clone 53-6.7, BioLegend), CD69 (clone H1.2F3, BioLegend), CD134 (clone H1.2F3, BioLegend), and T-bet (clone 4B10, BD). For intracellular cytokine staining or intracellular molecule phosphorylation staining, cells were restimulated for 4 to 6 hours with phorbol 12-myristate 13-acetate (50 ng/ml) (Sigma-Aldrich, MO, USA), ionomycin (1 μg/ml) (Sigma-Aldrich, MO, USA), and brefeldin A (3 μg/ml) (eBioscience, CA, USA). Intracellular cytokines were stained using the intracellular fixation/permeabilization buffer set (eBioscience, CA, USA). Samples were analyzed on a CytoFLEX LX flow cytometer (Beckman Coulter) and analyzed with FlowJo software (version 10.0.7; Tree Star).

### Western blot

Cells were collected and lysed with cell lysis buffer in the presence of protease inhibitor phenylmethylsulfonyl fluoride (PMSF) for 30 min on ice. Protein concentration was determined by the BCA protein assay kit (EMD Millipore Corp., Billerica, MA, USA) according to the manufacturer’s instruction. Equal amounts of protein from each cell lysate were subjected to 10% or 12% SDS–polyacrylamide gel electrophoresis (SDSPAGE). The resolved proteins were transferred to polyvinylidene difluoride membranes and blotted with indicated Abs, anti-HA, anti-FLAG, anti-Myc, anti–TREM-2, anti-CD3ζ, anti-ZAP70, anti–p-CD3ζ, or anti–p-ZAP70. Actin or β-tubulin was used as an internal control. Then, the membranes were incubated with appropriate secondary Abs at room temperature for 1 hour and lastly visualized on GE ImageQuant LAS 500 using an ECL kit (Fdbio Science). The quantitative intensity analysis of Western blot bands has been performed with ImageJ software.

### Immunoprecipitation

Plasmids containing HA-tagged TREM-2 full-length, Ig domain, or truncated forms of TREM-2 respectively deleting transmembrane (△TM), cytosolic domain (△Cyto), or Ig domain (△Ig) Myc-tagged CD3ζ were generated by molecular clone. FLAG-tagged SARS-CoV-2 M protein, FLAG-tagged SARS-CoV-2 N protein, and FLAG-tagged SARS-CoV-2 RBD were provided by P. Wang’ s laboratory (Shandong University, China). Indicated constructs were transfected into HEK293T cells using Lipofectamine 2000 for 48 hours. Cellular lysates were prepared by incubating the cells in cell lysis buffer in the presence of protease inhibitor PMSF for 30 min on ice, followed by centrifugation at 13,000 rpm for 10 min at 4°C. For IP, cellular or lung tissue lysates were incubated with anti-FLAG (Sigma-Aldrich) or HA M2 affinity gel (Sigma-Aldrich) or consecutively incubated with control (IgG) or TREM-2 Abs (clone D418C, CST) and protein A/G agarose beads (EMD Millipore) for 12 hours at 4°C with constant rotation. Beads were then washed eight times using the cell lysis buffer. Between washes, the beads were collected by centrifugation at 13,000 rpm for 1 min at 4°C. The precipitated proteins were eluted from the beads by resuspending the beads in 5× SDSPAGE loading buffer and boiling for 10 min. The boiled immune complexes were subjected to SDSPAGE, followed by immunoblotting with appropriate Abs.

### Pseudovirus/lentivirus production

The gene encoding M membrane glycoprotein and human TREM-2 was synthesized by BGI (Beijing, China). The pseudovirus of SARS-CoV-2 M was generated from VSV expression system, which was replaced the glycoprotein gene (G) with the M protein of SARS-CoV-2 (*39*, *40*). Briefly, the gene encoding M membrane glycoprotein was cloned into the lentiviral expression plasmid pcDNA3.1, and an mCherry tag was added. Pseudovirus expression vector (pcDNA3.1-M-mCherry and pcDNA3.1–TREM-2–GFP), pHIV-GFP-luc expression vector, pgagpol HIV vector, pHIV-Rev, and pHIV-TAT were cotransfected into HEK293T cells with 70 to 80% cell density in a 100-mm dish. To produce lentivirus containing human TREM-2, the gene encoding human TREM-2 was cloned into the lentiviral expression plasmid pCDH, and a GFP tag was added. Vectors were produced by standard transient transfection of a three-plasmid system into HEK293T cells. Briefly, 10 μg of lentiviral expression vector (pcDH-M-mCherry), 7.5 μg of packaging plasmid ps-PAX2, and 2.5 μg of envelope plasmid pMD2.G were transfected to HEK293 cells with 70 to 80% cell density in a 100-mm dish using polyethylenimine. Culture medium was replaced with Dulbecco’s modified Eagle’s medium (DMEM) [10% fetal bovine serum (FBS)] 10 hours after transfection. Pseudoviral-containing supernatants were collected 24 and 48 hours after transfection, filtered, pooled, and mixed with polyethylene glycol 8000 at 4°C overnight and then concentrated by centrifugation at 4000*g* for 30 min. Pseudovirus vector titer was determined by a quantitative real-time PCR-based method to detect stably integrated virus sequences (copy number) in target HEK293T cells and was expressed as transducing units per milliliter.

### Cell sorting and culture

Human CD4^+^ and CD8^+^ T cells were sorted from PBMCs of healthy donors and patients with COVID-19 using the magnetic cell sorting system (BD). The purity of isolated cells was confirmed as more than 95%. Cells were cultured in 10% FBS containing RPMI 1640 medium. To study the effect of COVID-19 plasma on TREM-2 expression on T cells, sorted CD4^+^ T cells from healthy donors were stimulated with plasma from heterogeneous healthy donors and patients with COVID-19; the ratio of medium to plasma was 20:1. To obtain TREM-2–overexpressed CD4^+^ T cells, lentivirus containing TREM-2 was added to the culture system for 12 hours. To study the effect of TREM-2–Fc fusion protein, PP2, or SH-4-54 on T cell response, human CD4^+^ T cells and CD8^+^ T cells were pretreated with TREM-2–Fc fusion protein (300 ng/ml; 1828-T2, R&D Systems), TREM-1–Fc fusion protein (300 ng/ml; 1278-TR, R&D Systems), TREM-2 Ab (1 μg/ml; clone 237920, R&D Systems), or isotype IgG (300 ng/ml; 110-HG, R&D Systems), PP2 (250 nM), or SH-4-54 (100 nM) versus dimethyl sulfoxide (DMSO) for 1 hour. Cells were then stimulated with pseudovirus of SARS-CoV-2 M protein, recombinant SARS-CoV-2 M protein (1 μg/ml; Wksubio, China), or anti-CD3 agonist Ab (1 μg/ml; clone OKT3, BD) for the indicated times and collected for flow cytometric analysis and Western blot.

### Solid phase binding assay

The procedure for cell staining in this study was described according a previous study (*34*) with some modification. A 96-well plate was coated with TREM-2–Fc or TREM-1–Fc or control IgG (2 μg/ml) (R&D Systems) in PBS overnight at 4°C. After washing and blocking with 3% BSA in PBS for 1 hour at 37°C, recombinant M protein (Wksubio, China) was diluted as indicated into a concentration in PBS containing 0.5% BSA or PS/SM (Avanti Polar Lipids) diluted into a concentration of 10 μg/ml in methanol, was added, and incubated for 20 min at 37°C. After washing, the binding of M protein/Fc proteins were detected with biotinylated anti–M protein Ab (Abnova) for 1 hour at 37°C. Plates were washed and then incubated with avidin–horseradish peroxidase (Solarbio) for 30 min at 37°C, washed again, developed with TMB substrate solution (Solarbio), and read at 450 nm.

### MHV-A59 infection of mice

MHV-A59 C57BL/6 was propagated using the L929 cell line, and the titer of the virus was determined by plaque assay in L929 cells exactly as described. WT mice were purchased from the Guangdong Medical Laboratory Animal Center. The TREM-2–floxed mice were crossed with CD4-cre transgenic mice (Model Animal Research Center Co. Ltd.) to produce *TREM-2^fl/fl^Cd4*-Cre (CD4–TREM-2 KO) mice. Mice were intranasally inoculated with 20 μl of MHV-A59 [10^5^ plaque-forming units (PFU)], and control mice were inoculated intranasally with an equal volume of DMEM. We found that mice developed severe progressive pulmonary disease by days 2 to 3 after MHV-A59 infection with 100% mortality within 5 to 7 days of infection for CD4–TREM-2 cKO mice. CT imaging of each mice were gained on a small animal positron emission tomography (PET) imaging system (nanoScan PET/CT 82s) at 3 days after infection. At 5 days after infection, mice were euthanized. Plasma of mice were collected by centrifugation at 3000 rpm for 5 min for ELISA assay, and lungs were separated into three portions for measurements of viral load by plaque assays or PCR and for histological analysis. The surface activation markers in lung-infiltrating T cells were determined by flow cytometry. Intracellular molecule phosphorylation staining was performed as above and determined by flow cytometry. All experiments were performed with protocols approved by the Laboratory Animal Center, the Fifth Affiliated Hospital of Sun Yat-sen University.

### Enzyme-linked immunosorbent assay

Cytokines were quantified by commercially available ELISAs for IFN-γ and TNF-α according to the manufacturer’s instruction (R&D Systems). sTREM-2 in supernatants of the lung tissue was quantified by a commercial kit according to the manufacturer’s instruction (MultiSciences).

### Viral load by plaque assay

The lung tissue of infected mice and control uninfected mice were weighed and triturated in DMEM (containing 10% FBS) and rapidly frozen and thawed for their times. Cell debris was removed by centrifugation, and the virus titers (PFU per gram of tissue) in the supernatants were determined by plaque assay on L929 cells. Briefly, L929 cells were deposited in 12-well dishes, and supernatants of the lung tissue homogenate were added in each well (300 μl), three dilutions of 10^−1^, 10^−2^, and 10^−3^ were set, respectively. The supernatants containing virus were replaced with DMEM (containing 1% methyl cellulose and 2.5% FBS) after incubating at 37°C for 2 hours. Cells were cultured for another 3 days, and plaques were clearly visualized by crystal violet dyeing (1%).

### Histopathology evaluation

Lung tissue from the mice and patients with COVID-19 were fixed in zinc formalin. For routine histology, sections were stained with hematoxylin and eosin (Servicebio, Wuhan, China), and the pathology of the lung was evaluated under a microscope (Olympus BX53, Japan).

### Statistical analysis

Data analyses were performed in GraphPad Prism 5.0 Software (San Diego, CA). Statistical significance was determined with Kruskal-Wallis test or Mann-Whitney test for nonparametric tests, as well as with analysis of variance (ANOVA) or Student’s *t* test analyses for parametric tests. Data are shown as means ± SD unless otherwise stated. A *P* value of <0.05 was regarded as statistically significant.

## Notes

View/request a protocol for this paper from *Bio-protocol*.
