# Supplementary material for: TREM-2 is a sensor and activator of T cell response in SARS-CoV-2 infection
Source: Sci Adv. 2021 Dec 8;7(50):eabi6802. doi: 10.1126/sciadv.abi6802 (PMC8654301; doi:10.1126/sciadv.abi6802)
Supplement: Supplementary file 1 — Figs. S1 to S19 Tables S1 to S4 [file sciadv.abi6802_sm.pdf]

Science Advances
AAAS

Supplementary Materials for

TREM-2 is a sensor and activator of T cell response in SARS-CoV-2 infection

Yongjian Wu, Manni Wang, Huan Yin, Siqi Ming, Xingyu Li, Guanmin Jiang, Ye Liu, Peihui Wang, Guangde Zhou, Lei Liu, Sitang Gong, Haibo Zhou, Hong Shan, Xi Huang*

*Corresponding author. Email: huangxi6@mail.sysu.edu.cn

Published 8 December 2021, *Sci. Adv.* **7**, eabi6802 (2021)
DOI: 10.1126/sciadv.abi6802

This PDF file includes:

Figs. S1 to S19
Tables S1 to S4

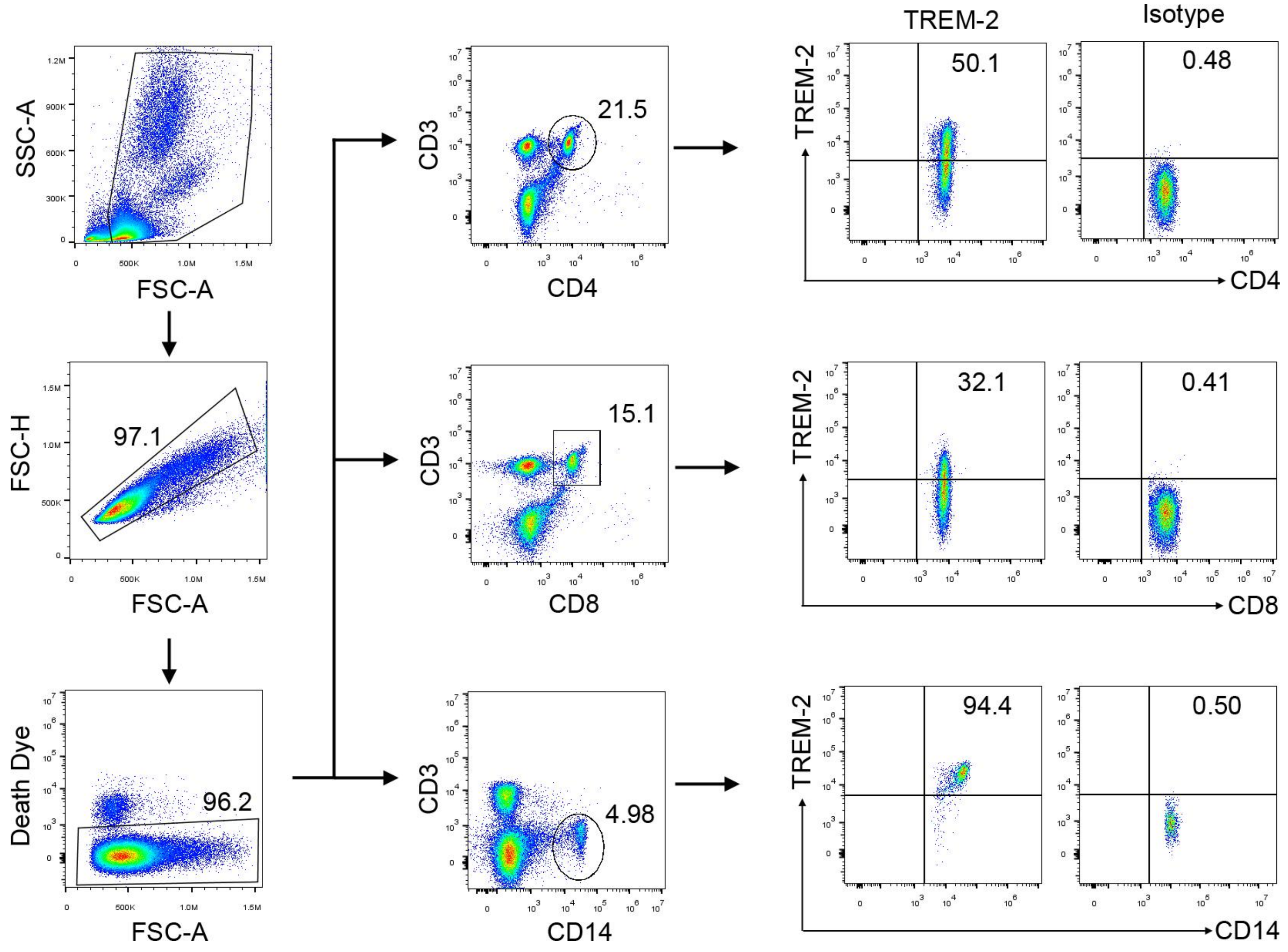

Fig. S1. Gate strategy related to Figure 1. Representative gating of $CD4^+$ T cells, $CD8^+$ T cells and $CD14^+$ monocytes from COVID-19 patient is shown. Briefly, mononuclear cells were gated out of all events followed by subsequent singlet gating. Live cells are gated as Death Dye^-. Cells were then gated as $CD3^+CD4^+$, $CD3^+CD8^+$ or $CD14^+CD3^-$ cells. The TREM-2 positive cell in these subsets was gated by staining with TREM-2 Ab and isotype control.

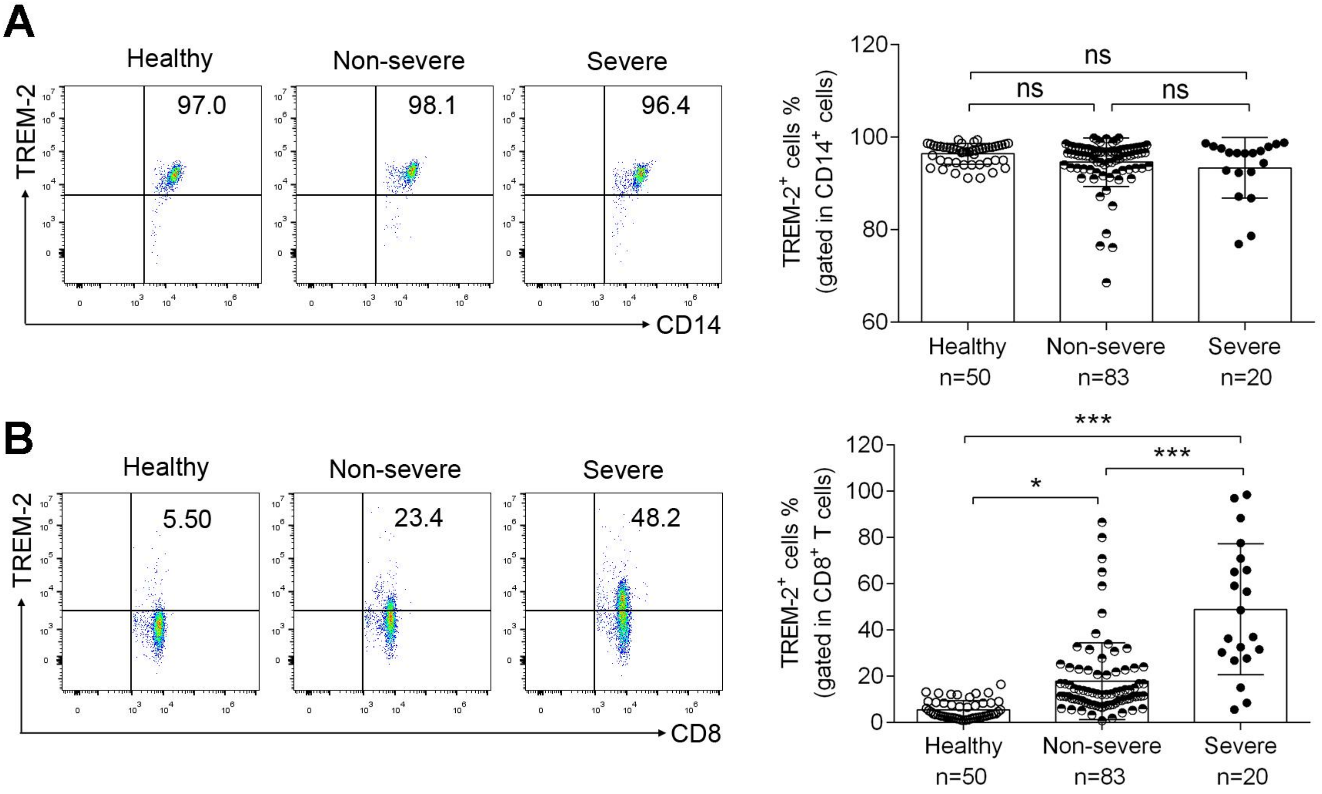

Fig. S2. The expression of TREM-2 on CD8^{+}T cells and CD14^{+}monocytes in COVID-19 patients. Flow cytometric analysis of TREM-2 expression in CD8^{+} T cells **(a)** and CD14^{+}monocytes **(b)** from healthy donors (n=50) or non-severe (n=83), and severe COVID-19 patients (n=20). ns, no significant. *P < 0.05, ***P < 0.001.

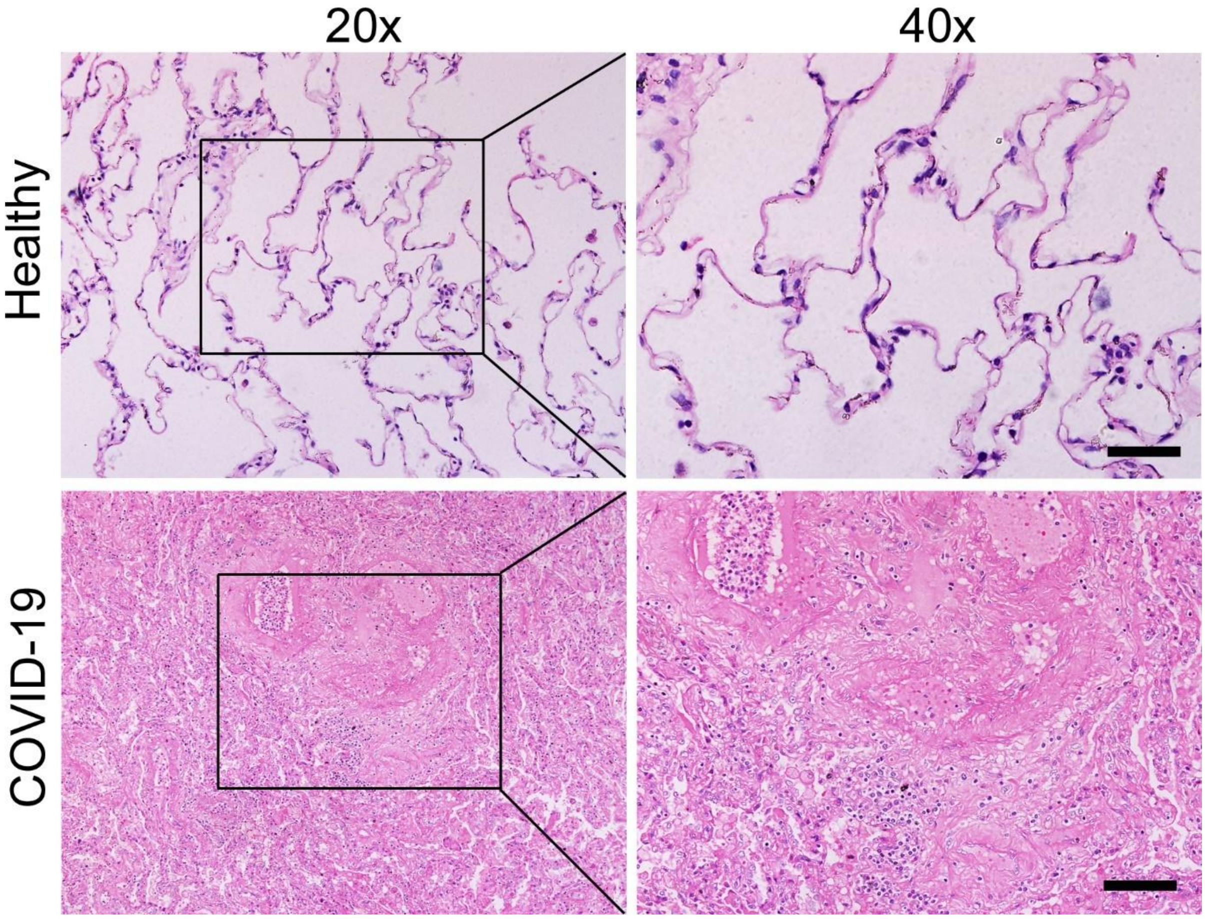

Fig. S3. Pulmonary histopathology associated with critical COVID-19 patient. Hematoxylin-and-eosin staining of normal lung tissues (defined as healthy) vs lung pathological sections from one critical COVID-19 patient. Scale bars, 50μm.

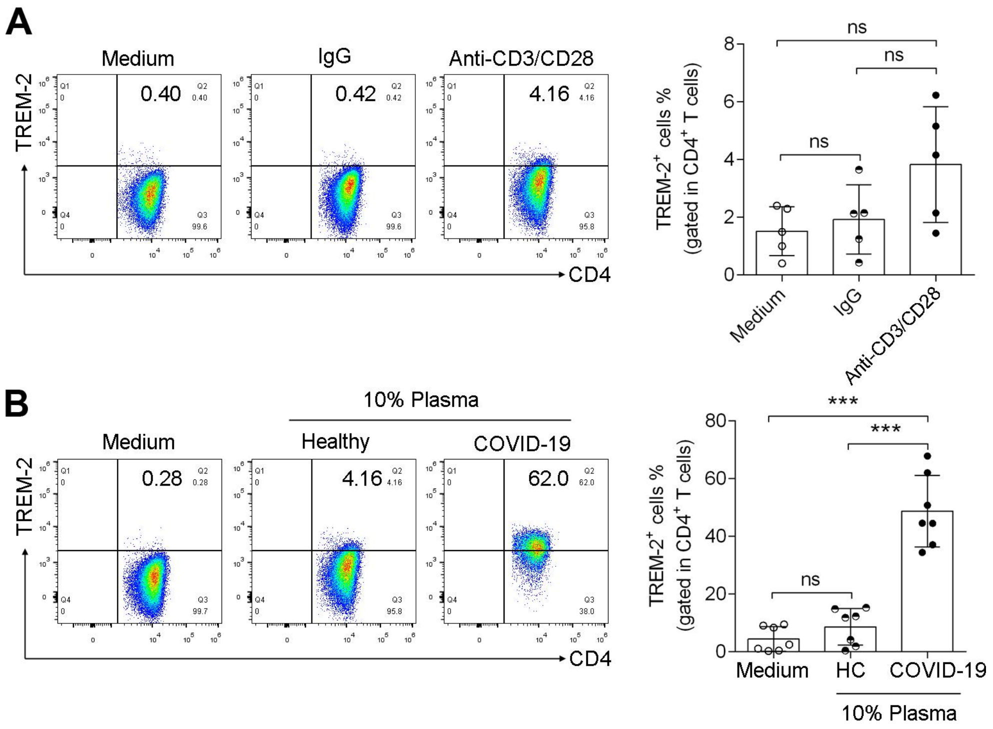

Fig. S4. COVID-19 plasma increased TREM-2 expression on CD4$^+$T cells. Sorted CD4$^+$T cells from healthy donors were treated with anti-CD3 Ab vs isotype IgG (**A**), healthy plasma vs COVID-19 plasma (**B**) for 24h. TREM-2 expression was determined by flow cytometry. ns, no significant. ***P < 0.001.

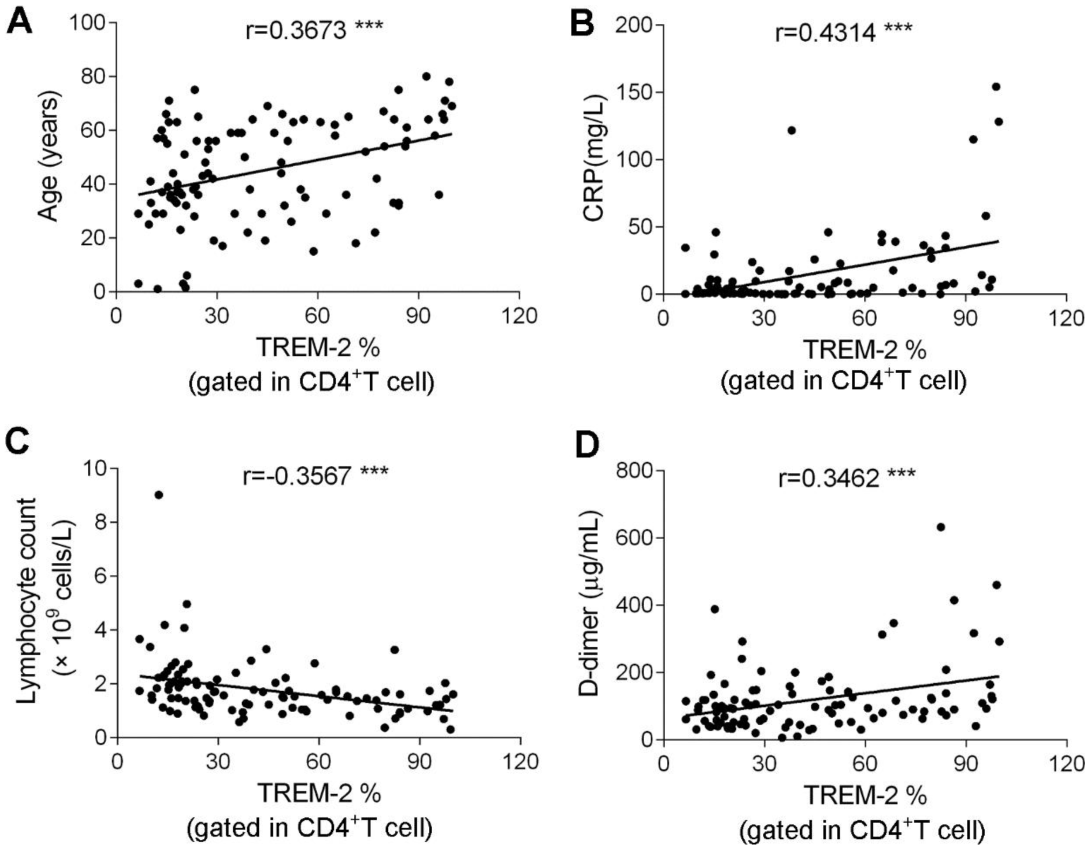

Fig. S5. TREM-2 expression on $CD4^+T$ cell was positively correlated with clinical indicators of severe COVID-19. Correlation between the frequency TREM-2^+CD4^+ T cells and Age of patients**(a)**, CRP level**(b)**, lymphocyte count**(c)**, D-dimer level**(d)** were analyzed in COVID-19 patients(n=103) by SPSS software. r, correlation coefficient. ***$P < 0.001$.

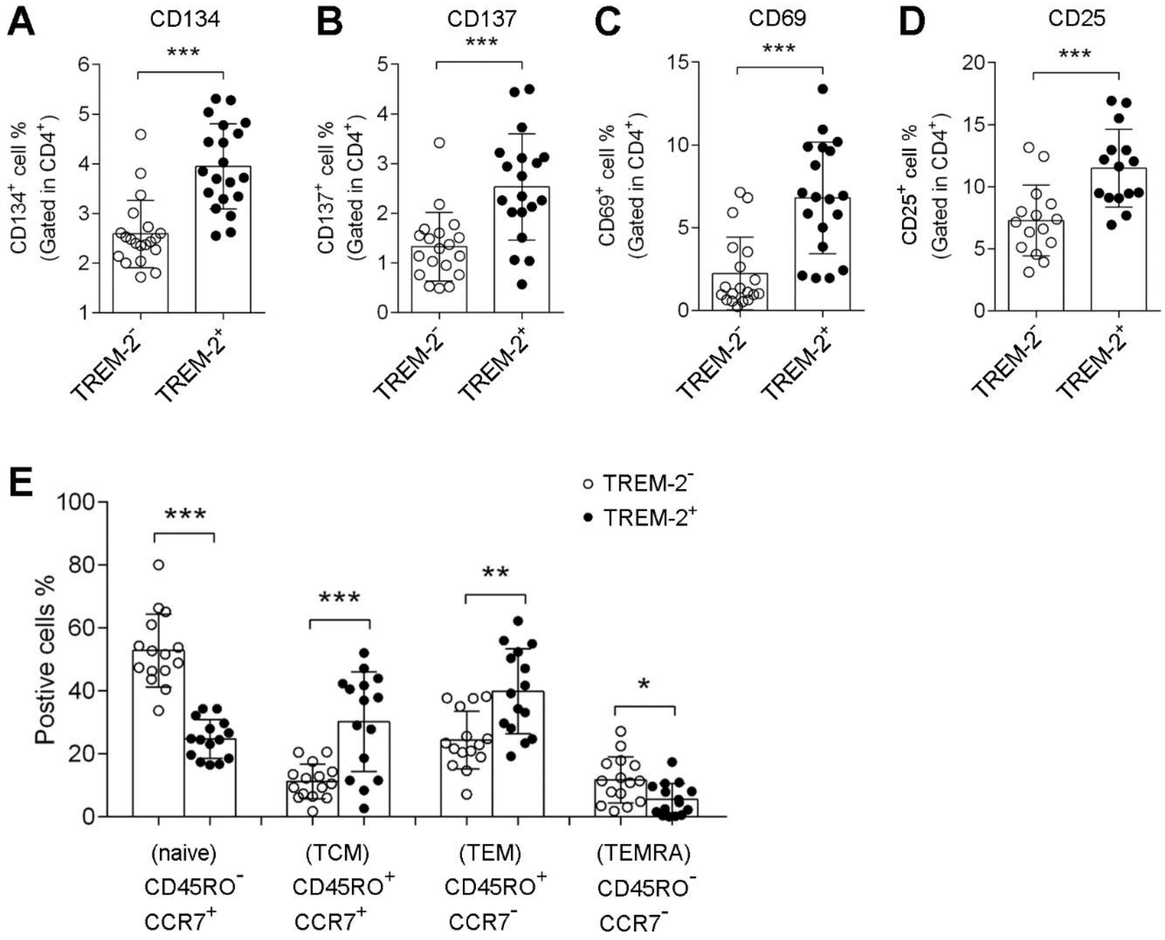

Fig. S6. TREM-2 expression was positively correlated with activation phenotype of T cells. (**a-d**) Expressions of T cell activation indicate markers including CD134 **(a)**, CD137 **(b)** and CD69 **(c)** as well as CD25 **(d)** were determined by flow cytometry. Percentages of positive cells for each indicate marker in TREM-2^{+} vs TREM-2^{-}CD4^{+}T cells from COVID-19 patients (n=20) were compared. (**e, f**) Flow cytometry analysis with CD45RO/CCR7staining to define the T cell subsets in TREM-2^{+} vs TREM-2^{-}CD4^{+}T cells from COVID-19 patients (n=15). Percentages of naive T cells, TCM, TEM and terminally differentiated effector cells TEMRA were compared in TREM-2^{+} vs TREM-2^{-} CD4^{+}T cells. Data are representative of three independent experiments. *$P < 0.05$, **$P < 0.01$, ***$P < 0.001$

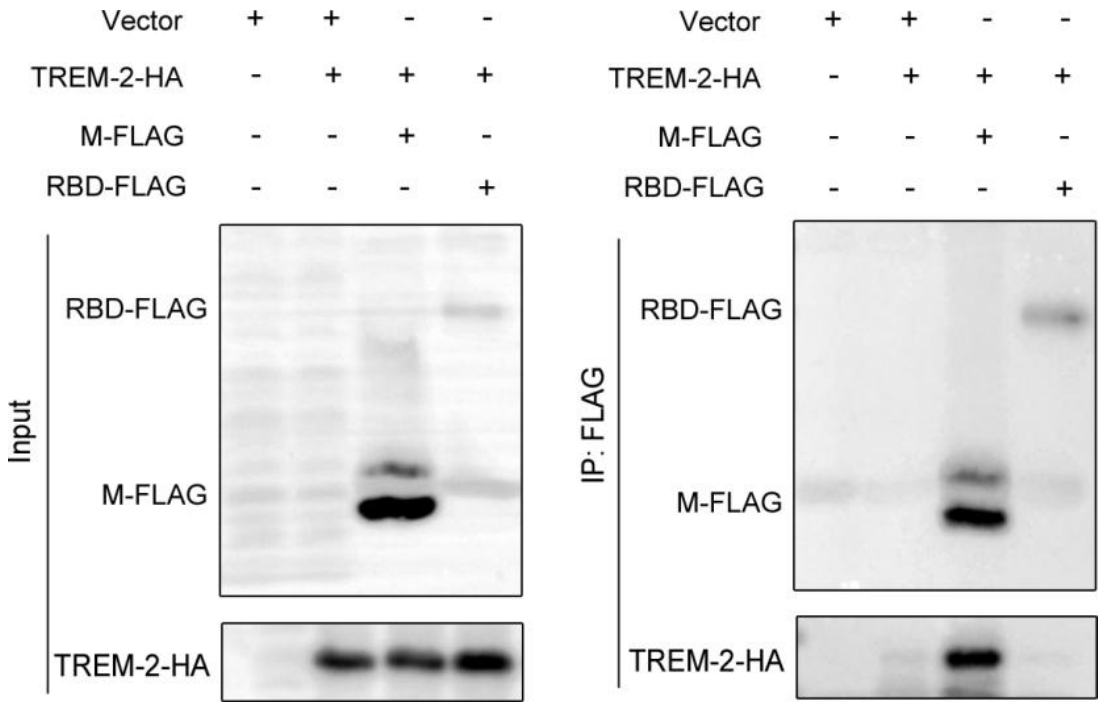

Fig. S7. TREM-2 did not interact with RBD domain of SARS-CoV-2. HEK293T cells were transfected with plasmids containing HA-tagged TREM-2 and FLAG-tagged SARS-CoV-2 RBD protein. Blots of cell lysates (input) and immunoprecipitation of anti-FLAG (IP: FLAG).

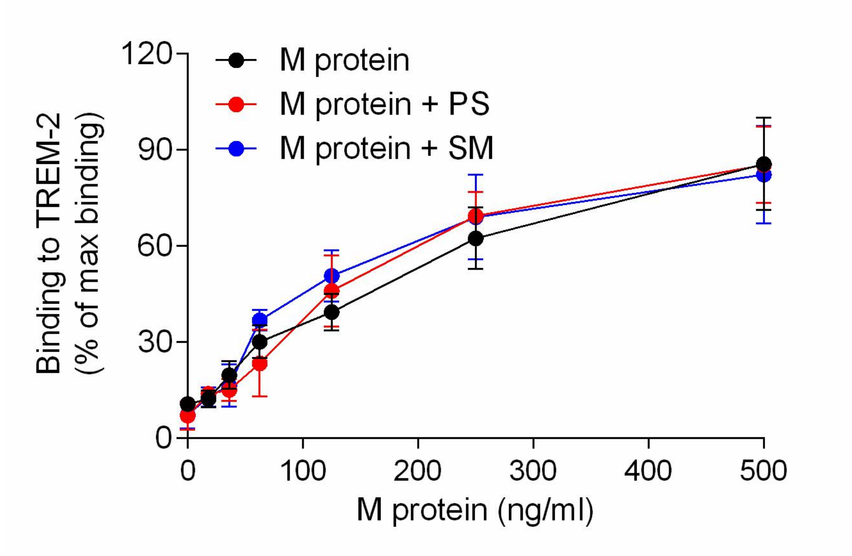

Fig. S8. PS or SM did not affect the binding of M protein and TREM-2. The plate was coated with TREM-2-Fc fusion protein, and then recombinant M protein combined with PS or SM was added. The bound Fc proteins were detected with anti-M protein antibody. The binding rate of M protein/TREM-2 was normalized to the binding of M protein/its antibody.

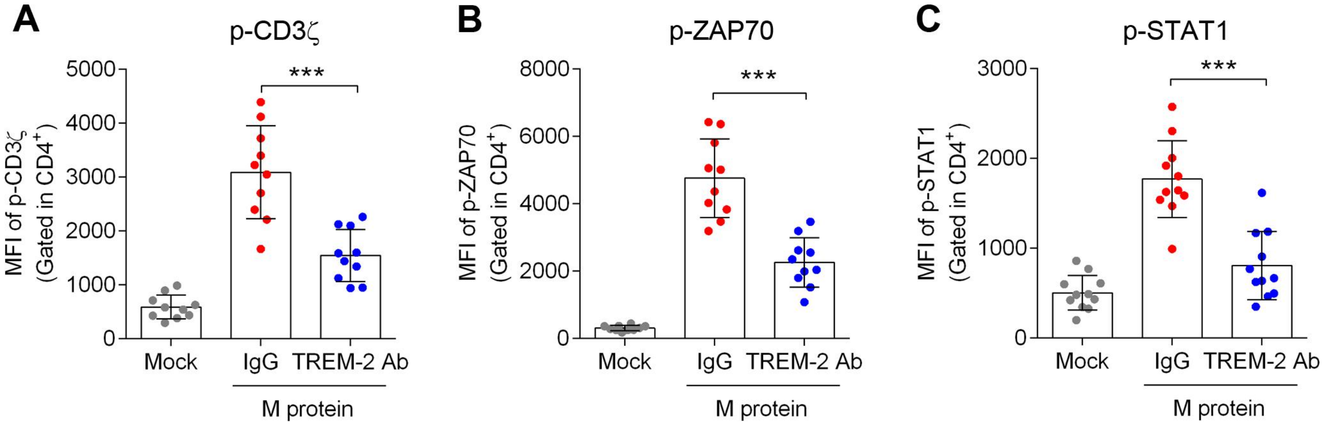

Fig. S9. TREM-2 antibody reduced the activation of CD3ζ/ZAP70/STAT1 signal pathway in M protein stimulated CD4$^+$T cells. Sorted CD4$^+$ T cells from COVID-19 patients (n=10) were cultured and stimulated with recombinant M protein in the presence of TREM-2 antibody or isotype IgG for 12 hours, and no stimulation (Mock) were as control. MFI of p-CD3ζ **(A)**, p-ZAP-70 **(B)** and p-STAT1 **(C)** in CD4$^+$ T cells were analyzed by flow cytometry. ***$P < 0.001$

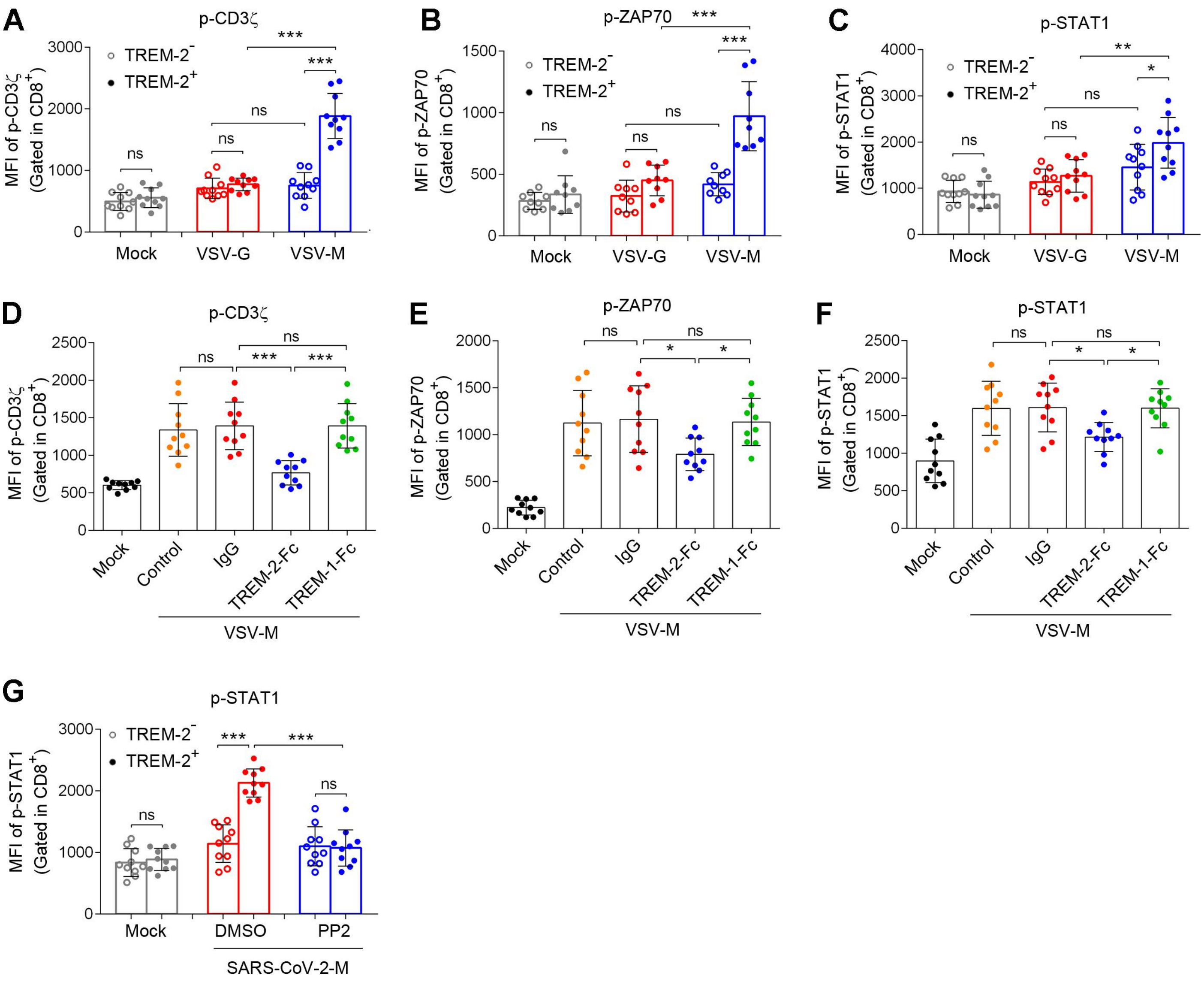

Fig. S10. TREM-2 enhanced CD3ζ/ZAP70/STAT1 signal pathway in SARS-CoV-2 M protein stimulated CD8$^+$T cell. (A-C) Sorted CD8$^+$ T cells from COVID-19 patients (n=10) were cultured and stimulated with pseudovirus of SARS-CoV-2 M protein (VSV-M) for 12 hours, and no stimulation (Mock) and empty pseudovirus vector (VSV-G) stimulation were as control. MFI of p-CD3ζ **(A)**, p-ZAP-70 **(B)** and p-STAT1 **(C)** -producing cells in TREM-2$^-$ vs TREM-2$^+$ CD8$^+$ T cells were analyzed by flow cytometry. MFI of p-CD3ζ **(D)** , p-ZAP-70 **(E)** and p-STAT1 **(F)** in CD8$^+$T cells in the presence of TREM-2-Fc or TREM-1-Fc fusion protein or isotype IgG for 12 hours, and no stimulation (Mock) and only pseudovirus of SARS-CoV-2 M protein stimulation (Control) were as control. **(G)** Flow cytometric analysis of p-STAT1 MFI in TREM-2$^-$ vs TREM-2$^+$ CD8$^+$ T cells in the presence of ZAP-70 inhibitor PP2 or DMSO. ns, no significant. *$P < 0.05$, **$P < 0.01$, ***$P < 0.001$

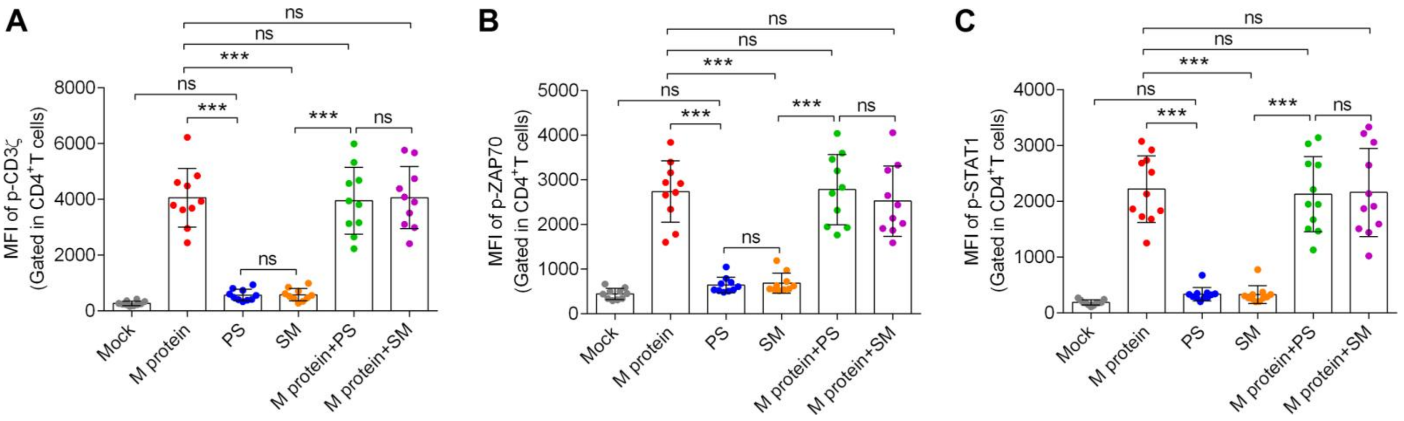

Fig. S11. Lipid component did not induce the activation of CD3ζ/ZAP70/ STAT1 signal pathway. Sorted CD4^{+}T cells from COVID-19 patients (n=10) were cultured and stimulated with M protein or PS or SM for 12 hours. MFI of p-CD3ζ **(A)**, p-ZAP-70 **(B)** and p-STAT1 **(C)** in CD8^{+}T cells were analyzed by flow cytometry. ns, no significant. ***P < 0.001.

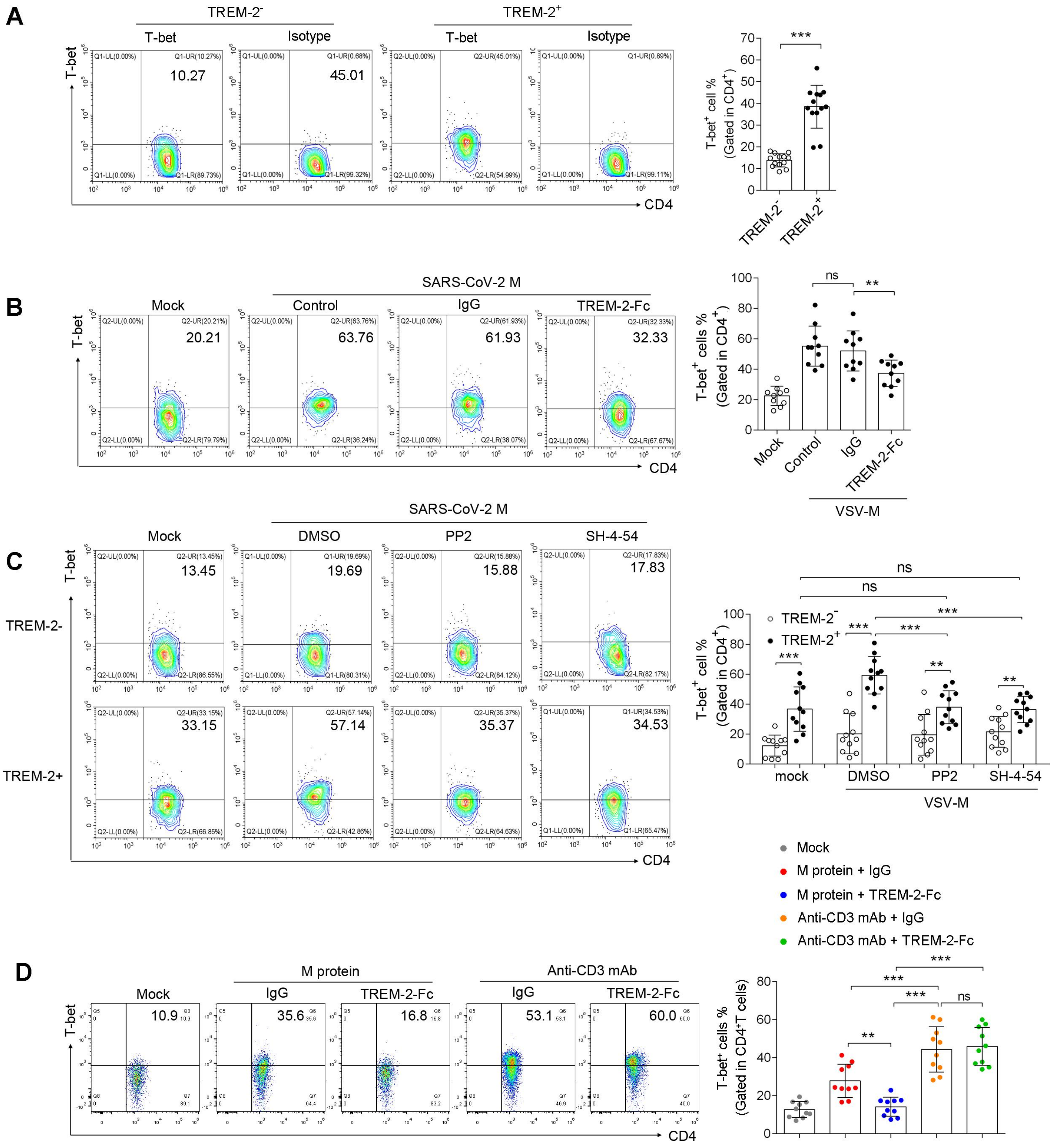

Fig. S12. TREM-2 enhanced T-bet expression in SARS-CoV-2 M protein stimulated CD4+T cell. (A) The intracellular T-bet in TREM-2$^-$ vs TREM-2$^+$CD4$^+$T cell from COVID-19 patients was determined by flow cytometry. **(B)** Sorted CD4$^+$ T cells from COVID-19 patients (n=10) were cultured and stimulated with pseudovirus of SARS-CoV-2 M protein in presence of TREM-2-Fc fusion protein or isotype IgG for 12 hours, and no stimulation (Mock) and only pseudovirus of SARS-CoV-2 M protein stimulation (control) were as control. **(C)** Sorted CD4$^+$T cells from COVID-19 patient were stimulated with pseudovirus of SARS-CoV-2 M protein in the presence of ZAP-70 inhibitor PP2, Pan STATs inhibitor SH-4-54 or DMSO.

(**D**) Sorted $CD4^{+}$T cells from COVID-19 patient were stimulated with recombinant M protein or anti-CD3 antibody in the presence of TREM-2-Fc fusion protein or isotype IgG for 12 hour. Flow cytometric analysis of T-bet expression in $TREM\text{-}2^{-}$ vs $TREM\text{-}2^{+}$ $CD4^{+}$ T cells from COVID-19 patients. ns, no significant. $^{**}P < 0.01$, $^{***}P < 0.001$.

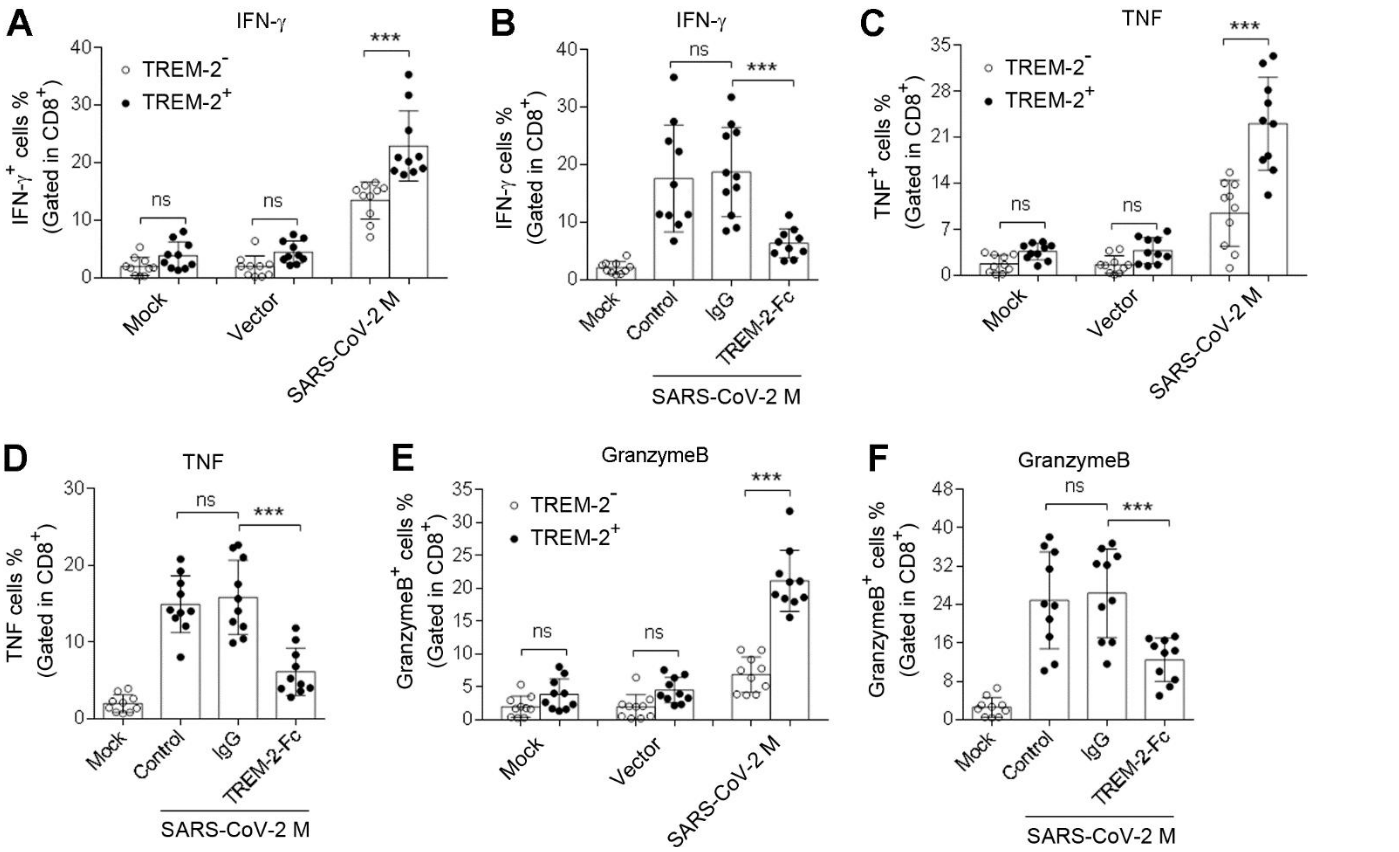

Fig. S13. TREM-2 facilitated Th1 cytokines and GranzymeB production in $CD8^+$ T cells in COVID-19 patients. (a, c, e) Sorted $CD8^+$T cells from COVID-19 patients (n=10) were cultured and stimulated with pseudovirus of SARS-CoV-2 M protein for 12 hours, and no stimulation (Mock) and empty pseudovirus vector (Vector) stimulation were as control. **(b, d, f)** Sorted $CD8^+$T cells from COVID-19 patients (n=10) were cultured and stimulated with pseudovirus of SARS-CoV-2 M protein in presence of TREM-2-Fc fusion protein or isotype IgG for 12 hours, and no stimulation (Mock) and only pseudovirus of SARS-CoV-2 M protein stimulation (control) were as control. And then Cells were stimulated with PMA, ionomycin and BFA for 6 hours. Percentages of IFN-γ, TNF-α and GranzymeB-producing cells in $TREM\text{-}2^-$ vs $TREM\text{-}2^+CD8^+$T cells were analyzed by flow cytometry. ns, no significant. *$P < 0.05$, ***$P < 0.001$.

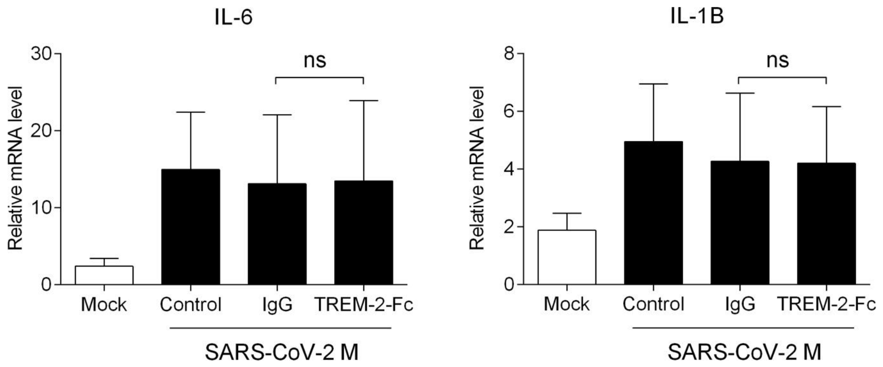

Fig. S14. TREM-2 did not affect the mRNA expression of IL-6 and IL-1 in SARS-CoV-2 M protein stimulated $CD4^{+}$T cell. Sorted $CD4^{+}$T cells from COVID-19 patients (n=10) were cultured and stimulated with pseudovirus of SARS-CoV-2 M protein in presence of TREM-2-Fc fusion protein or isotype IgG for 12 h, and no stimulation (Mock) and only pseudovirus of SARS-CoV-2 M protein stimulation (control) were as control. The expression level of IL-6(Left) and IL-1β(Right) were analyzed by real-time PCR. ns, no significant.

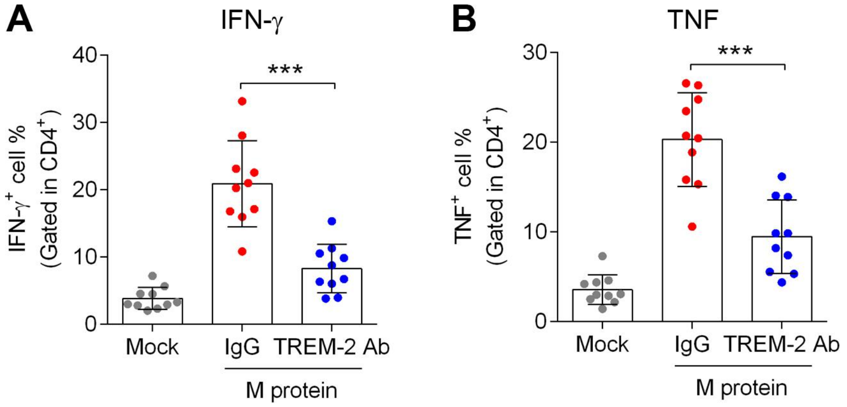

Fig. S15. TREM-2 antibody inhibited M protein-induced Th1 cytokines production in $CD4^+$ T cells . Sorted $CD8^+$T cells from COVID-19 patients (n=10) were cultured and stimulated with M protein for 12 hours. And then Cells were stimulated with PMA, ionomycin and BFA for 6 hours. Percentages of IFN-γ and TNF -producing cells in $CD4^+$T cells were analyzed by flow cytometry. ***$P < 0.001$.

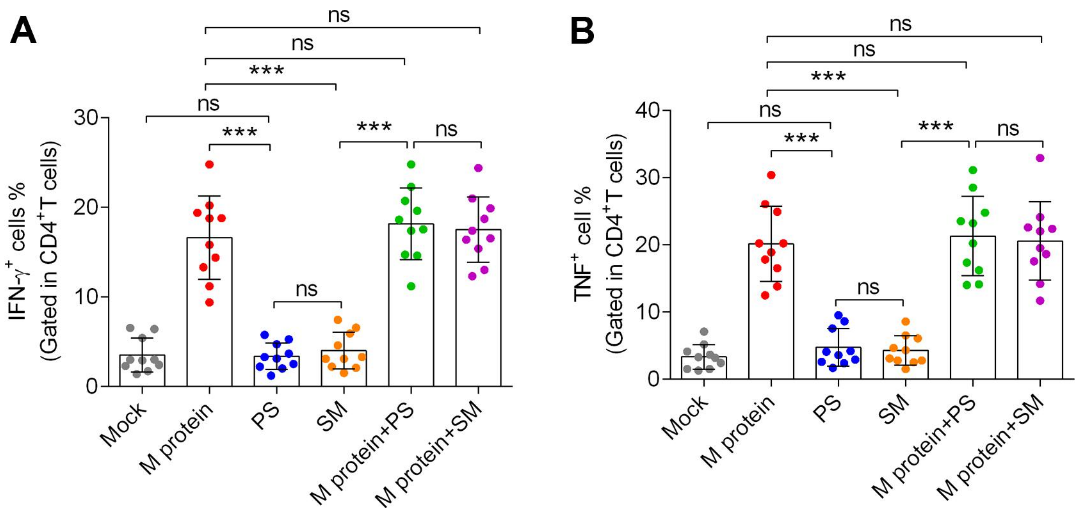

Fig. S16. Lipid did not promote M protein-induced Th1 cytokine production in $CD4^{+}T$ cells. Sorted $CD4^{+}T$ cells from COVID-19 patients (n=10) were cultured and stimulated with M protein or PS or SM for 12 hours. And then Cells were stimulated with PMA, ionomycin and BFA for 6 hours. Percentages of IFN-γ and TNF-producing cells in $CD4^{+}T$ cells were analyzed by flow cytometry. ns, no significant. ***$P < 0.001$.

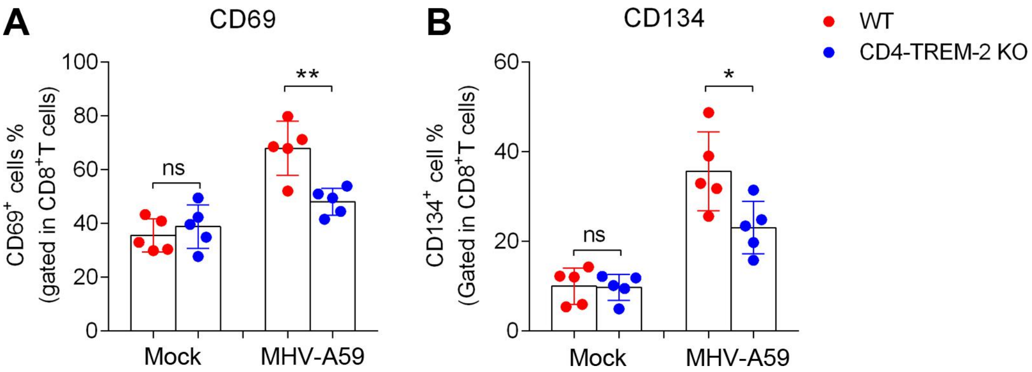

Fig. S17. Deficiency of TREM-2 in CD4⁺T cell reduced the activation phenotype of CD8⁺T cell in MHV-A59 infection *in vivo*. The surface expression of CD69 and CD134 in the lung was analyzed in MHV-A59 infected WT mice vs CD4-TREM-2 KO mice by flow cytometry. The uninfected mice were as control (Mock). ns, no significant. $*P < 0.05$, $**P < 0.01$.

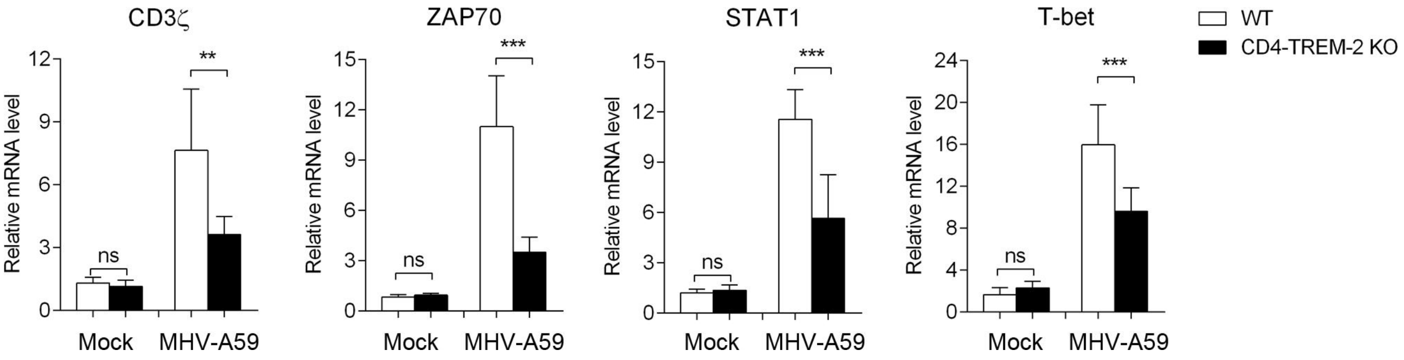

Fig. S18. Deficiency of TREM-2 in CD4⁺T cell impaired the CD3ζ/ZAP70/ STAT1/T-bet signal during MHV-A59 infection *in vivo*. The mRNA expression in the lung for CD3ζ, ZAP70, STAT1 and T-bet were analyzed in MHV-A59 infected WT mice vs CD4-TREM-2 KO mice by real-time PCR. The uninfected mice were as control (Mock). ns, no significant. $^{**}P < 0.01$, $^{***}P < 0.001$.

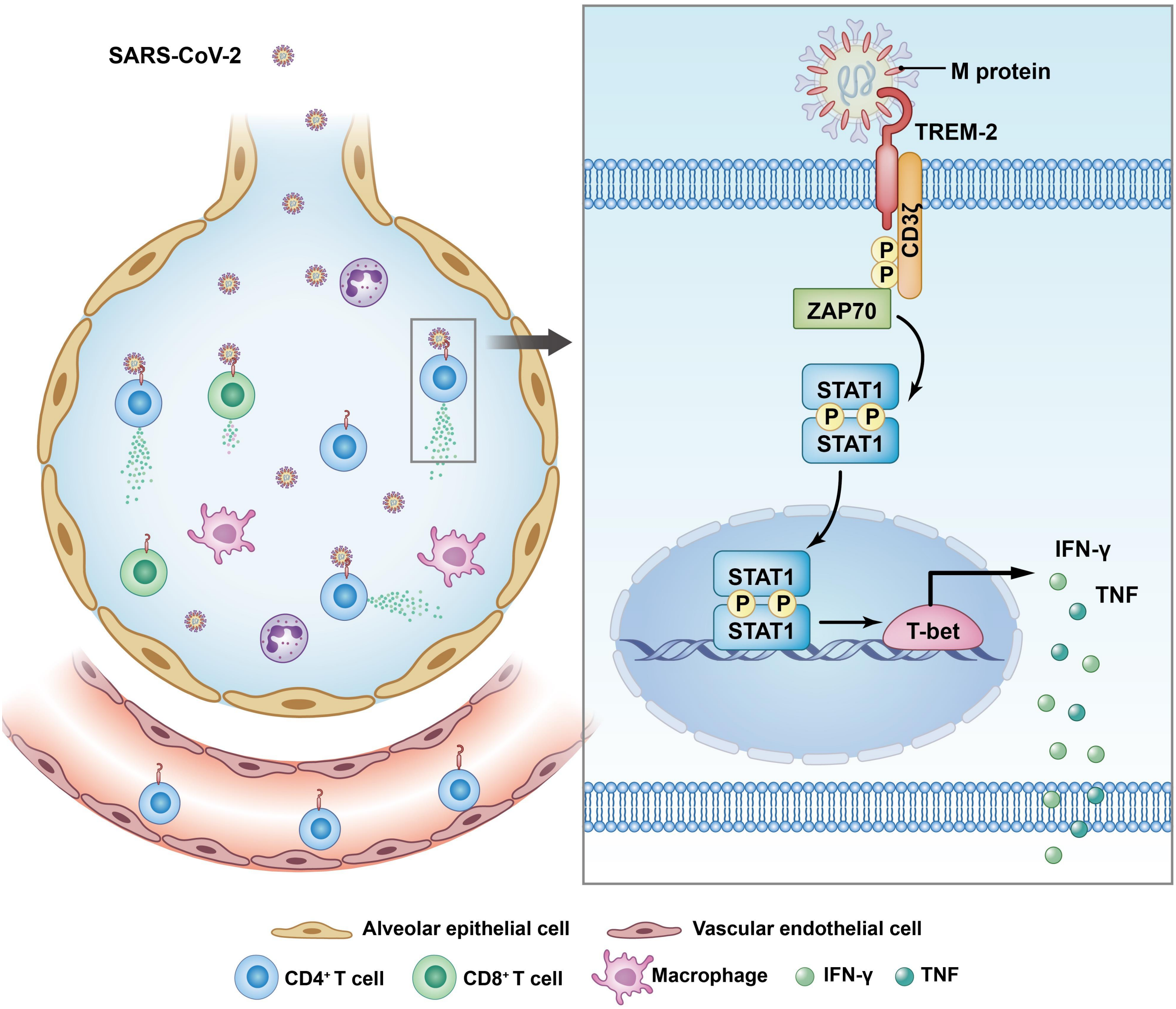

Fig. S19. Graphical Abstract. After SARS-CoV-2 infection, TREM-2 is upregulated in peripheral and lung infiltrating T cell, resulting in pro-inflammatory Th1 responses during the acute phase of COVID-19. In this process, TREM-2 interacts with SARS-CoV-2 membrane protein with its Ig domain. The activation of TREM-2 recruits CD3ζ/ZAP70 complex to induce the STAT1/T-bet signaling pathways, which leads to promote the secretion of Th1 cytokines including IFN-γ and TNF.

Table S1: Summary of clinical features and laboratory results of SARS-CoV-2 patients					
	Healthy donor n=50	Non-severe n=93	Severe n=20	*P* value Non-severe vs Severe	Reference Value
Age (years)	43.39 ± 2.12	41.57 ± 1.95	59.00 ± 3.24	< 0.0001	N.A.
Sex (male/female)	21/29	36/47	12/8	0.2167	N.A.
Interval between diagnosis and sample collection (days)	N.A.	1.39 ± 1.19	1.05 ± 0.89	0.237	N.A.
Interval between admission to hospital and sample collection (days)	N.A.	2.01 ± 1.76	2.45 ± 1.79	0.315	N.A.
Symptoms and signs					
Fever	N.A.	47(50.54%)	18(90.0%)	N.A.	N.A.
Cough	N.A.	39(41.94%)	11(55.0%)	N.A.	N.A.
Generalised weakness	N.A.	3(3.22%)	7(35.0%)	N.A.	N.A.
Nasal congestion	N.A.	16(17.20%)	5(25.0%)	N.A.	N.A.
Rhinorrhoea	N.A.	10(10.75%)	3(15.0%)	N.A.	N.A.
Sore throat	N.A.	16(17.20%)	5(25.0%)	N.A.	N.A.
Dyspea	N.A.	0(0%)	5(25.0%)	N.A.	N.A.
Nausea or vomiting	N.A.	10(10.75%)	0(0%)	N.A.	N.A.
Headache	N.A.	7(7.53%)	1(5.0%)	N.A.	N.A.
Myalgia	N.A.	10(10.75%)	2(10.0%)	N.A.	N.A.
Diarrhoea	N.A.	7(7.53%)	3(15.0%)	N.A.	N.A.
Body temperature (°C)	36.8± 0.07	37.30 ± 0.09	37.76 ± 0.16	0.0222	36-37
Oximetry saturation (%)	N.A.	97.57 ± 0.35	96.41 ± 0.42	0.1099	93-98
Haemoglobin (g/dL)	N.A.	138.4 ± 1.90	133.8 ± 4.51	0.2987	13.3-17.1 (male); 11.5-14.8 (female)
White blood cell count (× 10^9 cells/L)	N.A.	5.49 ± 0.25	5.07 ± 0.39	0.4464	3.9-9.9
Neutrophil count (× 10^9 cells/L)	N.A.	2.935 ± 0.16	3.47 ± 0.41	0.1687	2.0-7.4
Lymphocyte count (× 10^9 cells/L)	N.A.	1.94 ± 0.13	1.09 ± 0.09	0.0014	1.1-3.6
Platelet count (× 10^9 cells/L)	N.A.	223.5 ± 8.26	154.6 ± 8.41	0.0001	162-341

Monocyte count (× 10^9 cells/L)	N.A.	0.55 ± 0.03	0.50 ± 0.05	0.4468	0.1-0.6
Prothrombin time (s)	N.A.	11.97 ± 0.11	12.57 ± 0.31	0.0282	11.0-14.5
International normalised ratio	N.A.	1.05 ± 0.01	1.11 ± 0.03	0.0114	0.8-1.15
Activated partial thromboplastin time (s)	N.A.	31.31 ± 0.34	30.94 ± 1.01	0.6663	26.0-40.0
D-dimer (ug/mL)	N.A.	102.5 ± 9.95	176.0 ± 27.44	0.0031	0.0-0.5
Fibrinogen (g/dL)	N.A.	2.97 ± 0.084	3.75 ± 0.21	0.0002	2.0-4.0
C-reactive protein (mg/L)	N.A.	9.08 ± 2.39	39.06 ± 9.07	< 0.0001	0.0-5.0
Albumin (g/L)	N.A.	40.46 ± 0.37	36.91 ± 0.76	< 0.0001	35.0-52.0
Bilirubin (μmol/L)	N.A.	7.84 ± 0.43	9.035 ± 0.96	0.2348	0.0-21.0
Alanine aminotransferase (U/L)	N.A.	21.47 ± 1.73	21.91 ± 3.04	0.9095	0.0-33.0
Aspartate aminotransferase (U/L)	N.A.	21.50 ± 0.90	26.63 ± 2.56	0.0221	0.0-32.0
Urea (mmol/L)	N.A.	3.639 ± 0.14	5.03 ± 1.01	0.0157	2.8-8.1
Creatinine (μmol/L)	N.A.	59.65 ± 2.17	82.50 ± 15.15	0.0089	44-80
Lactate dehydrogenase (U/L)	N.A.	171.0 ± 5.10	218.3 ± 15.98	0.0004	135-214
Sodium (mmol/L)	N.A.	140.0 ± 0.30	137.1 ± 0.51	< 0.0001	136-145
Potassium (mmol/L)	N.A.	3.762 ± 0.04	3.69 ± 0.09	0.4472	3.5-5.1
Creatine kinase (U/L)	N.A.	79.76 ± 5.08	126.6 ± 43.53	0.0486	0-170

Table S1. Summary of clinical features and laboratory results of SARS-CoV-2 patients

Patient NO.	Gender	Age	Symptoms When Admitted	Virus nucleci	Subgroups	Sample	Clinical history	Comorbidities
1	Male	66	High fever (39°C), had cough for 8 days	Positive	critical type	Lung tissue (right, 1 sample), pathological section	The patient had a history of hypertension for 8 years and no diabetes, bronchiectasis, tuberculosis and other diseases.	Acute respiratory failure, acute respiratory distress syndrome (ARDS), septic shock, and other severe complications
2	Male	62	head trauma	Negative (unexposed)	N/A	Lung tissue (right, 1 sample)	N/A	N/A

Table S2. Overview of the characteristics of patient who studied for lung tissue.

Table S3. Protein identified by LC-MS mass spectrometry.		
prot_hit_num	prot_acc	prot_desc
1	sp|P63104|1433Z_HUMAN	14-3-3 protein zeta/delta OS=Homo sapiens OX=9606 GN=YWHAZ PE=1 SV=1
2	sp|Q5QNW6|H2B2F_HUMAN	Histone H2B type 2-F OS=Homo sapiens OX=9606 GN=HIST2H2BF PE=1 SV=3
3	sp|P07996|TSP1_HUMAN	Thrombospondin-1 OS=Homo sapiens OX=9606 GN=THBS1 PE=1 SV=2
4	sp|A0A075B6R9|KVD24_HUMAN	Probable non-functional immunoglobulin kappa variable 2D-24 OS=Homo sapiens OX=9606 GN=IGKV2D-24 PE=5 SV=1
5	sp|P06312|KV401_HUMAN	Immunoglobulin kappa variable 4-1 OS=Homo sapiens OX=9606 GN=IGKV4-1 PE=1 SV=1
6	sp|A0A075B6K5|LV39_HUMAN	Immunoglobulin lambda variable 3-9 OS=Homo sapiens OX=9606 GN=IGLV3-9 PE=3 SV=1
7	sp|P62805|H4_HUMAN	Histone H4 OS=Homo sapiens OX=9606 GN=H4C1 PE=1 SV=2
8	sp|P61626|LYSC_HUMAN	Lysozyme C OS=Homo sapiens OX=9606 GN=LYZ PE=1 SV=1
9	sp|P01876|IGHA1_HUMAN	Immunoglobulin heavy constant alpha 1 OS=Homo sapiens OX=9606 GN=IGHA1 PE=1 SV=2
10	sp|Q9NZC2|TREM2_HUMAN	Triggering receptor expressed on myeloid cells 2 OS=Homo sapiens OX=9606 GN=TREM2 PE=1 SV=1
11	sp|P31025|LCN1_HUMAN	Lipocalin-1 OS=Homo sapiens OX=9606 GN=LCN1 PE=1 SV=1
12	sp|Q96HU8|DIRA2_HUMAN	GTP-binding protein Di-Ras2 OS=Homo sapiens OX=9606 GN=DIRAS2 PE=1 SV=1
13	sp|A0A0A0MRZ8|KVD11_HUMAN	Immunoglobulin kappa variable 3D-11 OS=Homo sapiens OX=9606 GN=IGKV3D-11 PE=3 SV=6
14	sp|P0DP03|HVC05_HUMAN	Immunoglobulin heavy variable 3-30-5 OS=Homo sapiens OX=9606 GN=IGHV3-30-5 PE=3 SV=1
15	sp|P00966|ASSY_HUMAN	Argininosuccinate synthase OS=Homo sapiens OX=9606 GN=ASS1 PE=1 SV=2
16	sp|P37837|TALDO_HUMAN	Transaldolase OS=Homo sapiens OX=9606 GN=TALDO1 PE=1 SV=2
17	sp|P10809|CH60_HUMAN	60 kDa heat shock protein, mitochondrial OS=Homo sapiens OX=9606 GN=HSPD1 PE=1 SV=2
18	sp|P20963|CD3Z_HUMAN	CD247 molecule OS=Homo sapiens OX=9606 GN=CD247 PE=1 SV=1
19	sp|Q9BXM0|PRAX_HUMAN	Periaxin OS=Homo sapiens OX=9606 GN=PRX PE=1 SV=2
20	sp|Q86VD1|MORC1_HUMAN	MORC family CW-type zinc finger protein 1 OS=Homo sapiens OX=9606 GN=MORC1 PE=2 SV=2
21	sp|Q8NEZ3|WDR19_HUMAN	WD repeat-containing protein 19 OS=Homo sapiens OX=9606 GN=WDR19 PE=1 SV=2
22	sp|P59665|DEF1_HUMAN	Neutrophil defensin 1 OS=Homo sapiens OX=9606 GN=DEFA1 PE=1 SV=1
23	sp|P07900|HS90A_HUMAN	Heat shock protein HSP 90-alpha OS=Homo sapiens OX=9606 GN=HSP90AA1 PE=1 SV=5
24	sp|Q86SS6|SYT9_HUMAN	Synaptotagmin-9 OS=Homo sapiens OX=9606 GN=SYT9 PE=2 SV=1
25	sp|Q8N5H7|SH2D3_HUMAN	SH2 domain-containing protein 3C OS=Homo sapiens OX=9606 GN=SH2D3C PE=1 SV=1
26	sp|O75116|ROCK2_HUMAN	Rho-associated protein kinase 2 OS=Homo sapiens OX=9606 GN=ROCK2 PE=1 SV=4
27	sp|Q86XI8|ZSWM9_HUMAN	Uncharacterized protein ZSWIM9 OS=Homo sapiens OX=9606 GN=ZSWIM9 PE=1 SV=2

28	sp|Q8NEE6|DRC6_HUMAN	Dynein regulatory complex subunit 6 OS=Homo sapiens OX=9606 GN=FBXL13 PE=2 SV=3
29	sp|P06881|CALCA_HUMAN	Calcitonin gene-related peptide 1 OS=Homo sapiens OX=9606 GN=CALCA PE=1 SV=4
30	sp|Q5JWF2|GNAS1_HUMAN	Guanine nucleotide-binding protein G(s) subunit alpha isoforms XLas OS=Homo sapiens OX=9606 GN=GNAS PE=1 SV=2
31	sp|O75558|STX11_HUMAN	Syntaxin-11 OS=Homo sapiens OX=9606 GN=STX11 PE=1 SV=1
32	sp|P15153|RAC2_HUMAN	Ras-related C3 botulinum toxin substrate 2 OS=Homo sapiens OX=9606 GN=RAC2 PE=1 SV=1
33	sp|P25705|ATPA_HUMAN	ATP synthase subunit alpha, mitochondrial OS=Homo sapiens OX=9606 GN=ATP5F1A PE=1 SV=1
34	sp|Q7Z340|ZN551_HUMAN	Zinc finger protein 551 OS=Homo sapiens OX=9606 GN=ZNF551 PE=1 SV=3
35	sp|O76031|CLPX_HUMAN	ATP-dependent Clp protease ATP-binding subunit clpX-like, mitochondrial OS=Homo sapiens OX=9606 GN=CLPX PE=1 SV=2
36	sp|Q5R3F8|PPR29_HUMAN	Protein phosphatase 1 regulatory subunit 29 OS=Homo sapiens OX=9606 GN=ELFN2 PE=1 SV=1
37	sp|P14618|KPYM_HUMAN	Pyruvate kinase PKM OS=Homo sapiens OX=9606 GN=PKM PE=1 SV=4
38	sp|P05106|ITB3_HUMAN	Integrin beta-3 OS=Homo sapiens OX=9606 GN=ITGB3 PE=1 SV=2
39	sp|P09848|LPH_HUMAN	Lactase-phlorizin hydrolase OS=Homo sapiens OX=9606 GN=LCT PE=1 SV=3
40	sp|Q9BX97|PLVAP_HUMAN	Plasmalemma vesicle-associated protein OS=Homo sapiens OX=9606 GN=PLVAP PE=1 SV=1
41	sp|P24158|PRTN3_HUMAN	Myeloblastin OS=Homo sapiens OX=9606 GN=PRTN3 PE=1 SV=3
42	sp|A4D1F6|LRRD1_HUMAN	Leucine-rich repeat and death domain-containing protein 1 OS=Homo sapiens OX=9606 GN=LRRD1 PE=2 SV=2
43	sp|Q9H706|GARE1_HUMAN	GRB2-associated and regulator of MAPK protein 1 OS=Homo sapiens OX=9606 GN=GAREM1 PE=1 SV=2
44	sp|O94855|SC24D_HUMAN	Protein transport protein Sec24D OS=Homo sapiens OX=9606 GN=SEC24D PE=1 SV=2
45	sp|Q14728|MFS10_HUMAN	Major facilitator superfamily domain-containing protein 10 OS=Homo sapiens OX=9606 GN=MFSD10 PE=1 SV=1
46	sp|Q6I9Y2|THOC7_HUMAN	THO complex subunit 7 homolog OS=Homo sapiens OX=9606 GN=THOC7 PE=1 SV=3
47	sp|P49368|TCPG_HUMAN	T-complex protein 1 subunit gamma OS=Homo sapiens OX=9606 GN=CCT3 PE=1 SV=4
48	sp|Q5GJ75|TP8L3_HUMAN	Tumor necrosis factor alpha-induced protein 8-like protein 3 OS=Homo sapiens OX=9606 GN=TNFAIP8L3 PE=1 SV=1
49	sp|P61224|RAP1B_HUMAN	Ras-related protein Rap-1b OS=Homo sapiens OX=9606 GN=RAP1B PE=1 SV=1
50	sp|O00534|VMA5A_HUMAN	von Willebrand factor A domain-containing protein 5A OS=Homo sapiens OX=9606 GN=VWA5A PE=2 SV=2
51	sp|P43403|ZAP70_HUMAN	Zeta chain of T cell receptor associated protein kinase 70 OS=Homo sapiens OX=9606 GN=ZAP70 PE=1 SV=1
52	sp|O75197|LRP5_HUMAN	Low-density lipoprotein receptor-related protein 5 OS=Homo sapiens OX=9606 GN=LRP5 PE=1 SV=2
53	sp|Q6GYQ0|RGPA1_HUMAN	Ral GTPase-activating protein subunit alpha-1 OS=Homo sapiens OX=9606 GN=RALGAPA1 PE=1 SV=1
54	sp|Q86XX4|FRAS1_HUMAN	Extracellular matrix protein FRAS1 OS=Homo sapiens OX=9606 GN=FRAS1 PE=1 SV=2
55	sp|Q6ZUG5|YC006_HUMAN	Uncharacterized protein FLJ43738 OS=Homo sapiens OX=9606 PE=2 SV=1
56	sp|Q9UI10|EI2BD_HUMAN	Translation initiation factor eIF-2B subunit delta OS=Homo sapiens OX=9606 GN=EIF2B4 PE=1 SV=2

57	sp|A9Z1Z3|FR1L4_HUMAN	Fer-1-like protein 4 OS=Homo sapiens OX=9606 GN=FER1L4 PE=2 SV=1
58	sp|P60842|IF4A1_HUMAN	Eukaryotic initiation factor 4A-I OS=Homo sapiens OX=9606 GN=EIF4A1 PE=1 SV=1

Table S3. Mass spectrum identified protein interacted with TREM-2 in COVID-19 T cells

Deascription	Forward 5’-3’	Reverse 5’-3’
Human TREM-1	TGATCATGGTTTACTGCGCG	CTGGGGCTGGTATAGAGTGG
Human TREM-2	TGCGGAATCTACAACCCCAT	AGAAGGATGGAAGTGGGTGGT
Human TLT1	AGCAGCAGAGTTTCAGGCAT	ATGAGGATGGAGCTGGGAGT
Human TLT2	TGTCTGTGCAGTGCTCCTAT	ACAGGATCCCAGAGGTGTTG
Human TLT4	TCACAAACACCCAGGACAGA	CCGAGTCATTCTGTGTCAGC
Mouse IFN-γ	TTCAGCTCTGCATCGTTTTG	TCTTTTGGATGCTCTGGTCA
Mouse TNF-α	ATCAGAGGGCCTGTACCTCA	GGAAGACCCCTCCCAGATAG
Human IL-6	CACTCACCTCTTCAGAACGA	CAGGCAAGTCTCCTCATTGA
Human IL-1β	TGTAGTGGTGGTCGGAGA	GGCTGCCATGTCAGAAGA
MHV N gene	CAAAGAAAAGGGCGTAGACAGG	CGCCATCATCAA GGATCTGAGG
SARS-CoV-2 M	ATCCTTCGTGGACATCTTCG	TGCAGCAAAACCTGAGTCAC

Table S4. Primers used for current study
